# Multiple Arbuscular Mycorrhizal Fungal Consortia Enhance Yield and Fatty Acids of *Medicago sativa*: A Two-Year Field Study on Agronomic Traits and Tracing of Fungal Persistence

**DOI:** 10.3389/fpls.2022.814401

**Published:** 2022-02-14

**Authors:** Elisa Pellegrino, Marco Nuti, Laura Ercoli

**Affiliations:** ^1^Institute of Life Sciences, Scuola Superiore Sant’Anna, Pisa, Italy; ^2^University of Pisa, Pisa, Italy

**Keywords:** biofertilization, *Funneliformis mosseae*, *Rhizophagus irregularis*, microbial consortia, crop yield and quality, local and foreign AM fungal inocula, fatty acids (FAs)

## Abstract

Arbuscular mycorrhizal fungi are promoted as biofertilizers due to potential benefits in crop productivity, and macro- and microelement uptake. However, crop response to arbuscular mycorrhizal fungi (AMF) inoculation is context-dependent, and AMF diversity and field establishment and persistence of inoculants can greatly contribute to variation in outcomes. This study was designed to test the hypotheses that multiple and local AMF inoculants could enhance alfalfa yield and fatty acids (FA) compared to exotic isolates either single or in the mixture. We aimed also to verify the persistence of inoculated AMF, and which component of the AMF communities was the major driver of plant traits. Therefore, a field experiment of AMF inoculation of alfalfa (*Medicago sativa* L.) with three single foreign isolates, a mixture of the foreign isolates (FMix), and a highly diverse mixture of local AMF (LMix) was set up. We showed that AMF improved alfalfa yield (+ 68%), nutrient (+ 147% N content and + 182% P content in forage), and FA content (+ 105%). These positive effects persisted for at least 2 years post-inoculation and were associated with enhanced AMF abundance in roots. Consortia of AMF strains acted in synergy, and the mixture of foreign AMF isolates provided greater benefits compared to local consortia (+ 20% forage yield, + 36% forage N content, + 18% forage P content, + 20% total FA in forage). Foreign strains of *Funneliformis mosseae* and *Rhizophagus irregularis* persisted in the roots of alfalfa 2 years following inoculation, either as single inoculum or as a component of the mixture. Among inoculants, *F. mosseae* BEG12 and AZ225C and the FMix exerted a higher impact on the local AMF community compared with LMix and *R. irregularis* BEG141. Finally, the stimulation of the proliferation of a single-taxa (*R. irregularis* cluster1) induced by all inoculants was the main determinant of the host benefits. Crop productivity and quality as well as field persistence of inoculated AMF support the use of mixtures of foreign AMF. On the other hand, local mixtures showed a lower impact on native AMF. These results pave the way for extending the study on the effect of AMF mixtures for the production of high-quality forage for the animal diet.

## Introduction

The management of soil biota is considered a key strategy to maintain and improve ecological services in agro-ecosystems (De Vries et al., 2013; Toju et al., 2018). The plant symbiotic arbuscular mycorrhizal fungi (AMF) support plant growth, productivity, and soil fertility (e.g., Smith and Read, 2008; Gianinazzi et al., 2010). However, the evidence of crop benefits following field application of fungal inoculum suggests that intensive agricultural practices, such as frequent and deep tillage, high P or N fertilizer rate, long fallow, and continuous cropping, have a negative impact on AMF abundance in soil (e.g., Jasper et al., 1989; Lekberg and Koide, 2005; Roldan et al., 2007; Duan et al., 2010; Higo et al., 2013; Sheng et al., 2013; van der Heyde et al., 2017). In the European Union (EU), AMF are cataloged as plant biostimulants according to the new Regulation (EU) 2019/1009, based on the functions of stimulating plant nutrition processes and tolerance to abiotic stresses and improving the quality of the agricultural product (EU, 2019). AMF represent an important segment of the global biostimulant market, estimated in 2018 at about $ 2.0 billion (Rouphael and Colla, 2018). Despite the huge commercial interest in microbial and non-microbial plant biostimulants, associated with a growing body of research, the detailed molecular, cellular, and physiological mechanisms underlying plant-biostimulant interactions under different environments and management strategies remain largely unknown. Therefore, there is an urgent need to better elucidate the causal/functional mechanisms of biostimulants and their potential side-effects on the environment (e.g., invasiveness and threat to soil and plant biodiversity and ecosystem functioning, Hart et al., 2017).

In addition to the enhanced crop productivity (Lekberg and Koide, 2005; Pellegrino et al., 2011, 2015a; Pellegrino and Bedini, 2014), arbuscular mycorrhizal (AM) fungal addition through field inoculation can increase macro- and microelement content (Karandashov and Bucher, 2005; Veresoglou et al., 2012; Lehmann et al., 2014; Lehmann and Rillig, 2015; Ercoli et al., 2017a; Coccina et al., 2019; Pellegrino et al., 2020a) and nutraceuticals (Baslam et al., 2013; Saleh et al., 2020). Lekberg and Koide (2005), applying a meta-analysis approach on experimental results from field and glasshouse trials, demonstrated a large increase of mycorrhizal colonization in inoculation trials, suggesting that low inoculum potential can limit mycorrhizal colonization. Furthermore, they also showed that yield or biomass positive response to increased mycorrhizal colonization is non-linear, as management practices, site and climate, inherent inoculum potential, available soil P concentrations, and interactions between plant and fungal species modulate the plant response to increased mycorrhizal colonization.

In addition to abundance, AM fungal diversity can also limit AM fungal-derived benefits if different AM fungal species or isolates provide complementary benefits to the host plant (van der Heijden et al., 1998a,b; Verbruggen et al., 2010, 2013). Some pot studies with sterilized soil inoculated by a common bacterial community and AMF inocula have shown that plant growth and nutrient uptake were more promoted by diverse inocula, composed of a mixture of AM fungal taxa belonging to different species of the same family (Edathil et al., 1996; van der Heijden et al., 1998a,b) or to different families (Thonar et al., 2014; Crossay et al., 2019; Parvin et al., 2020), than single-species inocula. These results are supported by a meta-analysis of Hoeksema et al. (2010) in which AM fungal inoculum complexity (multispecies and whole soil inocula vs. single species inocula) was positively related to plant benefits. Conversely, other studies showed that pot inoculation with multiple AM fungal species could not outweigh single inoculants in terms of plant biomass response, while plant diversity and nutrient uptake [e.g., phosphorus (P) and nitrogen (N)] were even decreased (van der Heijden et al., 2003; Jansa et al., 2008). However, due to the different functional traits among AMF (Munkvold et al., 2004; Thonar et al., 2011; Treseder et al., 2018), and because the interactions among AMF are not always synergistic (Koide, 2000; Jansa et al., 2008), the effectiveness of AM fungal mixed inocula have to be evaluated in the field, where a local microbial community (including AMF and many other microbial groups) is present. However, when a crude inoculum is applied (composed of AM fungal spores, roots, and soil), the method utilized for the production of different isolates may include their associated microbial communities and metabolites that are likely to have an effect on the inoculation outcomes, thus representing a confounding effect (Bianciotto and Bonfante, 2002).

However, the effectiveness of AM fungal inocula should be confirmed by pieces of evidence of their establishment and persistence in the field. Attempts to directly track inoculants in the field are few due to the fact that morphological methods (i.e., staining and counting at the microscope) cannot be applied because many AM fungal species colonizing the same root are morphologically indistinguishable, while current molecular markers can hardly distinguish among closely related taxa and the detection of specific fungal species isolates remains challenging in the field (Hart et al., 2015). An earlier study, based on the amplification of ca. 2,200-bp-long central stretch of the nuclear rDNA cistron and the barcoding of SSU-ITS2-LSU sequences (550, 200, and 580 bp, respectively), successfully tracked the establishment and persistence of two isolates of *F. mosseae* (IMA1 = BEG12 and AZ22C) in alfalfa up to 2 years (Pellegrino et al., 2012). Similarly, other studies using different molecular targets and approaches [e.g., the LSU of ribosomal RNA gene or the mitochondrial LSU DNA (mtLSU) and cloning and sequencing; a fragment of the RNA polymerase II gene (RPB1) and qPCR; mt cox3-rnl intergenic mtDNA region, and droplet digital PCR] could detect root establishment and persistence of some AM fungal isolates in field-inoculated crops up to 3 years (Farmer et al., 2007; Sýkorová et al., 2012; Thioye et al., 2019).

Alfalfa (*Medicago sativa* L.) is a perennial herbaceous legume and one of the most important forage crops in the world due to its high feeding value and yield potential (Radović et al., 2009). Moreover, alfalfa supports important functions in sustaining agricultural systems, such as diversification of crop rotations, conserving soil water, improving soil structure by the development of a large root system, and increasing soil fertility through biological N fixation (Carlsson and Huss-Danell, 2003; Hakl et al., 2017; Sun and Li, 2019). Alfalfa’s global cultivation area exceeds 35 million ha (Radović et al., 2009) with an estimated world production of around 436 million tons in 2006 (FAO, 2006). Alfalfa contains a high quantity of macro- and microelements, and a high amount of fatty acids (FA), such as linoleic acid (C18:2) and the α-linolenic acid (C18:3) (Boufaïed et al., 2003) that are fundamental for animal health (Radović et al., 2009). The composition of fats from food animals is a major human health concern, and particularly important is the content of total polyunsaturated FA (ΣPUFA), α-linolenic acid (ALA, 18:3), and linoleic acid (LA, 18:2), ALA, and LA belonging to the n-3 (omega-3) and n-6 (omega-6) fatty acid family, respectively (Calviello and Serini, 2010). An increase of PUFAs and conjugated linoleic acids (CLA), such as ALA and LA in the human diet, has been shown to reduce the risk of cardiovascular disease, diabetes, cancers, and obesity (Pariza et al., 2001). Since animals cannot synthesize ALA or LA *de novo*, their content in forage plants, among which alfalfa is one of the main sources, is of primary importance in producing beef and milk with high levels of omega-3 FA and CLA (Dhiman et al., 1999; French et al., 2000; Scollan et al., 2001; Pintus et al., 2013).

To our knowledge, no information is available about the role played by AMF in plant FA synthesis, despite the fact that C in the mycorrhizal roots, received by the host plant in the form of monosaccharides, is stored primarily as lipids and many of the genes induced in mycorrhizal roots are predicted to function in lipid metabolism (Rich et al., 2017; Roth and Paszkowski, 2017). This study was designed to test the following multiple hypotheses: (i) alfalfa yield and quality is limited by AM fungal diversity and multiple AM fungal inoculants could act in synergy to promote crop growth and FA concentration; (ii) environmental pressure could select for highly efficient AM fungi and therefore a mixture of native AM fungal isolates could provide greater benefits compared to an exotic mixture. Finally, we aimed to verify the persistence of inoculated AMF in the field, and which component of the AM fungal communities found in the plots was the major driver of plant traits. To these aims, a field experiment of AM fungal inoculation of alfalfa with single foreign isolates, a mixture of the foreign isolates, and a highly diverse mixture of local AMF was set up and plants and fungal traits were assessed for 2 years, together with the molecular assessment of inoculum persistence and native AM fungal communities.

## Materials and Methods

### Fungal Material

The experiment compared five AM fungal inoculum treatments: (1) *Funneliformis mosseae* BEG 12, (2) *Funneliformis mosseae* AZ225C, (3) *Rhizophagus irregularis* BEG141, (4) a mixture of the above foreign isolates (Fmix) and (5) a mixture of local strains of AMF (Lmix). The Lmix was isolated from an alfalfa soil located in Manciano and was composed by *Funneliformis coronatum*, *Funneliformis geosporum*, *Funneliformis mosseae*, *Rhizophagus clarus*, *Rhizophagus irregularis*, *Glomus* sp., *septoglomus viscosum*, *Claroideoglomus etunicatum*, *Diversispora spurca*, *Acaulospora rugosa*, *Acaulospora cavernata*, *Acaulospora spinosa*, *Scutellospora aurigloba* and *Scutellospora calospora* (14 AM fungal species belonging to five families) (Pellegrino et al., 2020b). The foreign AMF isolates were obtained from the collection of the Scuola Superiore Sant’Anna, Italy. Details about the geographical origin, collector, and original inoculum supplier are given in Supplementary Table 1.

The inocula of single foreign AM fungal isolates (BEG12, AZ225C, BEG141) were produced in 15 L pots (four pots for treatment). Each pot was filled with sandy soil and Terragreen (1:1 by volume) and 1.5 L of starting crude inoculum. The substrate was previously steam-sterilized (121°C for 25 min, on two consecutive days) to kill native AMF. The Fmix was obtained at the end of inoculum production, by mixing equal quantities of the foreign AMF isolates. The Lmix was produced in 15 L pots (four pots), by adding 1.5 L soil of the experimental site to 13.5 L of the substrate. Additional four 15 L-pots were set up for the inoculation of control plots, by mixing equal quantities of sterilized single foreign AM fungal inocula and soil from the experimental site (a total of 1.5 L) to 13.5 L of the substrate (mock inoculum). Sudan grass (*Sorghum sudanense* L.) was used as a host plant (10 plants per pot). All pots received 1.5 L of a deionized water filtrate from the single foreign AM fungal inocula, and from the soil of the experimental site. Although the amount of microbial filtrate used at the start of the propagation was very large to minimize the differences among AM fungal inoculants, we cannot exclude that the differences in socialization among AM fungal isolates and microbes could lead to the end of the production phase to distinct microbial communities in the inoculants. Pots were irrigated with tap water and supplied with half-strength Hoagland’s solution every month (1.5 L per pot). After the 3-month growth, Sudan grass was harvested, and soil and roots were removed from the pots and air-dried. Then, the roots were cut, mixed with the soil, and stored in polyethylene bags at 4°C, until field inoculation. The AM fungal treatments were thus composed of AMF, microbes, soil, and roots, while the control was only composed of microbes, soil, and roots.

### Experimental Site and Climatic Data

The experiment was carried out at Manciano (Grosseto), Italy (47° 16′ 36.5′′ N–70° 14′ 53′′ E; 447 m above sea level). Main soil physical and chemical properties were: 322.9 g kg^–1^ sand, 308.5 g kg^–1^ silt, 368.6 g kg^–1^ clay (hydrometric method, Gee and Bauder, 1986); 7.1 pH (deionized H_2_O 1:2.5 w/v); 28.7 g kg^–1^ organic matter (Walkley–Black wet combustion method; Nelson and Sommers, 1982); 1.47 g kg^–1^ total N (Kjeldahl digestion; Bremner and Mulvaney, 1982);0.84 g kg^–1^ total P (colorimetry using perchloric acid digestion; Olsen and Sommers, 1982); 8.3 mg kg^–1^ available P (colorimetry using a solution of sodium bicarbonate; Olsen and Sommers, 1982); 163.2 mg kg^–1^ exchangeable K (Thomas, 1982); 30.1 meq 100 g^–1^ Cation Exchange Capacity (displacement with 0.1 M BaCl_2_ triethanolamine, Hendershot and Duquette, 1986). The soil of the area is a *Haplic Calcisol*, according to the FAO classification system (IUSS, 2015), and an Inceptisol, according to the USDA classification (Soil Survey Staff, 1999). The climate of the site is cold, humid Mediterranean (Csa) according to the Köppen-Geiger climate classification (Kottek et al., 2006). Mean annual maximum and minimum air temperatures averaged over 1996–2015 are 20.2 and 9.3°C, respectively, and annual precipitation was 769 mm (Vallebona et al., 2015). During the field experiment (November 2011–August 2013), maximum and minimum temperatures were 19.3 and 9°C, respectively, while total precipitation was 1,730 mm and had a peak of 207 mm during the 3rd decade of November 2012 (Supplementary Figure 1).

### Field Experiment Setup

A 2-year field AMF inoculation experiment was set up with alfalfa (*Medicago sativa* L., var. Giulia) as the host crop. Conventional tillage was performed at the beginning of September 2011 and consisted of moldboard plowing (30 cm depth), disking twice (15 cm depth), and harrowing (20 cm depth) (Ercoli et al., 2017b). Plots of 25 m^2^ size (5 m × 5 m) were designed within the field and hand inoculated with 0.4 kg m^–2^ of inoculum, incorporated into the soil by hand hoeing at ca. 10 cm depth (19 October 2011). The mean number of spores/sporocarps per g of inoculum ranged from 10 to 25, corresponding to 4,000–10,000 spores/sporocarps per m^2^. The plots were hand-seeded on 20 October 2011 with 5 g m^–2^ of alfalfa seeds. The preceding crop was barley. Neither chemical fertilization nor chemical nor mechanical weed control was applied. Alfalfa was cut when regrowth at the crown was initiated, on June 20 and August 6, 2012 and on 17 June and 18 August 2013. The experiment design was a nested split-plot with three replicates. The main plot factor was the age of cultivation (first and second year), the sub-plot factor was the AMF inoculation (BEG12, AZ225C, BEG141, Fmix, Lmix, and mock inoculum as control), and the cut was a nested factor within the year of cultivation.

### Sampling and Measurements

One month after crop emergence (December 2011), and at the beginning of spring growth in the two following years (2012 and 2013), six alfalfa plants from each replicated plot were sampled and AMF root colonization was assessed under a stereomicroscope (Olympus SZX 9, Olympus Optics, Tokyo, Japan), after clearing and staining with lactic acid instead of phenol (Phillips and Hayman, 1970), following the gridline intersect method (McGonigle et al., 1990). In both years and for both cuts, alfalfa shoots were harvested from one square meter for each replicated plot. A subsample of shoots was partitioned into stems and leaves, and dry weight was determined after oven drying at 75°C. Shoot N and P concentrations were determined by the Kjeldahl method and by the ammonium-molybdophosphoric blue color method, respectively (Jones et al., 1991). Protein concentration was calculated by multiplying the shoot N concentration × 6.25 (Lynch and Barbano, 1999). At the first cut of each year of cultivation, a root sample was extracted from the soil (about 100 g fresh weight) and assessed for AMF colonization applying the previously described procedure.

Fatty acids of alfalfa shoots from both cuts in the 2 years were extracted and methylated by a one-step procedure using toluene as solvent (Sukhija and Palmquist, 1988). Methyl non-adecanoate was used as an internal standard. Fatty acid methyl esters were quantified by gas chromatography using an HP 5890 chromatography (Hewlett Packard Co., Palo Alto, CA, United States), under the following conditions: 60-m × 0.32-mm DB-23 capillary column, 0.25 μm film thickness, H_2_ as a carrier gas, 2.8 cm^3^ min^–1^ volumetric flow rate, injector split 1/100 at 240°C, septum purge vent at 2 ml min^–1^, flame ionization detector at 250°C, and 15 kPa of heat pressure. The initial temperature was 150°C, which was increased by 5°C min^–1^ up to 200°C. Fatty acid methyl esters in toluene were directly injected through the split injection port. The peak area of Fas was measured using a Turbochrom 3 analytical system (version 3.3; PE Nelson, Cupertino, CA, United States). Each peak was identified and quantified using pure methyl ester standards (Alltech, Deerfield, IL, United States). Total volatile fatty acids were calculated.

### Extraction of Genomic DNA, PCR Amplification, Cloning, and Sequencing of Arbuscular Mycorrhizal Fungi Inoculants

In a further experiment, DNA was extracted from 50 spores of the foreign *F. mosseae* isolates BEG12 and AZ225C, from 20 spore clusters (ca. 25 spores each) of the foreign *R. irregularis* isolate BEG141 and from the roots of Sudan grass host plants grown in pots for the production of Lmix inoculum. Spores were crushed in microtubes on ice and the DNA was extracted using 50 μl of extraction buffer (100 mM Tris–HCl, 100 mM NaCl, 2 mM MgCl2, and 2% Triton-X100, pH 8). Genomic DNA was extracted from a subsample of 100-mg fresh weight of roots collected from the Sudan grass pots (four replicates), using the Dneasy^®^ Plant Mini Kit (Qiagen, Germantown, MD, United States). PCR amplification was performed using the primer pair NS31 and LSUGlom1, targeting a portion of the small subunit ribosomal RNA (SSU rRNA 18S) gene, the internal transcribed spacer (ITS1), the 5.8S, the ITS2, and a portion of the LSU rRNA gene (Pellegrino et al., 2012). The NS31/LSUGlom1 PCR amplicons were generated in volumes of 20 μl with 0.5 U of HotStarTaq DNA Polymerase (Qiagen, Germantown, MD, United States), 10 μM of each primer (NS31/LSUGlom1), 0.2 mM of each dNTP, 1 mM of MgCl_2,_ and 1 × reaction buffer, using a touchdown thermal cycling on an S1000 Thermal CyclerTM (BIORAD, Hercules, CA, United States). The temperature profile was as follows: denaturation and enzyme activation at 95°C for 15 min, 20 cycles with denaturation at 95°C for 30 s, primer annealing for 1 min starting at 62°C and decreasing by 0.5°C per cycle to 52°C, extension at 72°C for 135 s and 20 cycles with denaturation at 95°C for 30 s, primer annealing at 52°C for 1 min, extension at 72°C for 135 s, and a final extension at 72°C for 10 min. The QIAquick (Qiagen, Germantown, MD, United States) purified PCR amplicons of DNA from the spore samples and the Wizard^®^ SV (Promega Corporation, Madison, WA, United States) gel-purified amplicons of DNA from the root samples were ligated into the pGem®-T Easy vector (Promega, Corporation, Madison, WA, United States) to transform XL10-Gold^®^ Ultracompetent *Escherichia coli* cells (Agilent Technologies, Milano, Italy). On average, sixty-five recombinant clones per amplicon library were screened for the ca. 2,200-bp-long NS31-Glom1 fragment on agarose gels [2% ultrapure agarose (VWR) stained with 0.5 μg ml^–1^ ethidium bromide]. The variable SSU, ITS2, and LSU fragments of the vector inserts (2.2 kb long) were sequenced from plasmids, using the GenElute™ Plasmid Miniprep Kit (Sigma-Aldrich, St Louis, MO, United States). Sequencing reactions were set up with the vector primers SP6 and T7 using the 3730XL Genetic Analyser automated sequencer (Life Technologies, Carlsbad, CA, United States) at the DNA Services of the University of Illinois in Chicago, United States.

### Molecular Tracing of Arbuscular Mycorrhizal Fungi Inoculants in Roots of *M. sativa* Plants

At the onset of spring growth in 2013 (on the 2-year crop), 100 mg of fresh roots were collected from each replicate plot, and genomic DNA was extracted and amplified using NS31/LSUGlom1 primers, as described above. After screening on agarose, on average seventy recombinant clones were sequenced as described above. Plasmids (1184) were sequenced and phylogenetically identified targeting *F. mosseae* and *R. irregularis*, and other Glomeromycota taxa.

### Statistical Analysis

All results except for AM fungal root colonization were analyzed by a three-way ANOVA using a general mixed model including crop age (Age), AM fungal inoculant (Inoc), and cut (Cut) as fixed factors. Given our experimental design, the effect of the year of growth is completely confounded with the crop age and thus it was not possible to discriminate the effect of climatic conditions from the effect of crop age. Plots-within-replicates were included as a random factor to account for grouping factors and repeated measures in the same plots (Onofri et al., 2016). AM fungal root colonization was analyzed by a two-way ANOVA using a general mixed model and including Inoc and crop stage (1 month, 1st year, and 2nd year) as fixed factors and plots-within-replicates as a random factor. The relative abundances of AM fungal phylotypes (native *F. mosseae* and *R. irregularis*, and other Glomeromycota) in roots were analyzed by a one-way ANOVA to test the effect of Inoc. Data were ln or arcsine-transformed when needed to fulfill the assumptions of the ANOVA. *Post hoc* Tukey-B significant difference test was used for comparison among treatments. Finally, one-way ANOVA was performed to test the effect of inoculants on plant growth, N and P content of forage, and on single and total fatty acid content accumulated over cuts and years (data were transformed, when necessary, as described above). Differences between means were determined using orthogonal contrasts: inoculated (+ Myc) vs. control, single AM fungal isolates vs. mixture inoculants (Single vs. Mixture), and foreign vs. local AM fungal mixtures (Fmix vs. Lmix). The means given in tables and figures are for untransformed data. Analyses were performed using the SPSS software package version 21 (SPSS Inc., Chicago, IL, United States).

To understand the functional relationship between AM fungal community within the roots of alfalfa and plant performance, and which were the phylotypes mainly responsible for the measured plant traits, a two-step approach was utilized: (i) a multivariate descriptive analysis of the AM fungal community within the roots of the 2-year crop, and a multivariate descriptive analysis of the corresponding plant traits; (2) a test of the relationships between the biological community (AMF) and plant traits matrices. Thus, firstly, a non-metric multidimensional scaling analysis (nMDS) was done on the Bray-Curtis similarity matrix calculated on the fourth-root AM fungal relative abundances of phylotypes retrieved within the roots of inoculated and not-inoculated 2-year alfalfa (Kruskal, 1964). Moreover, the principal component analysis (PCA) was performed on the Euclidian distance matrix calculated on the square root of the plant traits (plant growth, N and P concentration of forage, and fatty acid composition) (Abdi and Williams, 2010). Then, a RELATE analysis, based on Spearman rank and 999 permutations, allowed to test the significance of the relationship between the two matrices (ρ = 1 perfect relationship) (Clarke and Warwick, 2001), while the BEST analysis, based on BioEnv methods (all combinations), Spearman rank and 999 permutations. Allowed to find the best descriptor of such relationship (Clarke et al., 2008). Finally, the Distance-based linear method (DistLM) analysis using a stepwise selection and the Akaike’s information criterion (AICc) was applied to measure the significance and the variance explained by the best descriptor/s (Knorr et al., 2000), and the Distance-based redundancy analysis was used to plot the first and second axes of the DistLM (Legendre and Anderson, 1999). Analyses were performed using PRIMER 7 and PERMANOVA + software (Primer-e, Auckland, New Zealand) (Anderson et al., 2008; Clarke and Gorley, 2015).

### Phylogenetic Analysis

The Glomeromycota affiliation of the sequences was verified in similarity searches using the Basic Local Alignment Search Tool (BLAST) in the National Center for Biotechnology Information (NCBI) database. No chimeric sequences were detected among the newly generated AM fungal SSU and ITS2-LSU sequences (1,324 sequences for each fragment). Here, 20 and 24 newly generated partial SSU (ca. 550 bp), ITS2 (ca. 165 bp) and LSU (ca. 400 bp) sequences of the isolates *F. mosseae* BEG12 and AZ225C, and 39 sequences of the native *F. mosseae* were aligned together with 17 public sequences of isolate BEG12 of *F. mosseae*, one sequence of the isolate *Funneliformis caledonium* BEG20, and one sequence of the isolate *Funneliformis geosporum* BEG11 using MAFFT online service (MAFFT version 7^1^; Kuraku et al., 2013; Katoh et al., 2019). Moreover, 17 newly generated partial SSU (ca. 720 bp), 5.8S and ITS2 (ca. 430 bp), and LSU (ca. 365 bp) sequences of the isolate *R. irregularis* BEG141, and 40 sequences of the native *R. irregularis* were aligned together with five sequences of the isolate *R. irregularis* DAOM181602, three sequences of the isolate *R. irregularis* MUCL43194/DAOM197198, four sequences of the isolate *R. irregularis* DAOM229456, and two sequences of the isolate *R. irregularis* MUCL41833, and one sequence of the isolate *R. intraradices* FL208. The multiple alignments were separately computed for the SSU and ITS2-LSU fragments and then concatenated using SEAVIEW^2^ (Gouy et al., 2010). Two phylogenetic trees (*F. mosseae* and *R. irregularis* trees) were built by neighbor-joining (NJ) analysis using built-in MEGA11^3^ (Tamura et al., 2021) and the Kimura 2-parameter model (Kimura, 1980). Branch support values correspond to 1,000 bootstrap replicates. The phylograms were drawn by MEGA 11 and edited by Adobe Illustrator 2021. Then, after having validated the DNA marker for the molecular discrimination among the foreign isolates of *F. mosseae* (12 and AZ225C) and *R. irregularis* (BEG141) and the native phylotypes of *F. mosseae* and *R. irregularis*, two further multiple sequence alignments were derived, including 478 and 332 newly generated sequences of *F. mosseae* and *R. irregularis*, respectively, derived from the roots sampled in the 2-year crop. Phylogenetic discrimination of non-native AM fungal strains relied on branch support of ≥ 70%. This approach of sequence analysis enabled to discriminate native from inoculated non-native strains through molecular barcoding and the phylogenetic divergence. The relative abundances of AM fungal intraradical communities (*F. mosseae* BEG12, AZ225C, BE141, native *F. mosseae*, native *R. irregularis*, and other Glomeromycota taxa) were calculated based on the ratio between the number of sequences affiliated to each phylotype and the total number of sequences of the clone library of each sample, and one-way ANOVA was performed to test the effect of inoculants and the *Post hoc* Tukey-B test was used for comparison among treatments. Cluster/Similarity Profile (SIMPROF) analyses were used to group the different samples into clusters based on their similarity/homogeneity of AM fungal intraradical communities (relative abundances of phylotypes) and to group the different phylotypes based on their similarity of occurrence. Relative abundance data were initially log(X + 1) transformed (Clarke and Warwick, 2001) and the Bray-Curtis similarity was calculated. The SIMPROF cluster analysis was performed to objectively define the groups within the dendrogram. Moreover, the relative abundances of the AM fungal phylotypes were represented by a shaded plot. The analyses were performed using PRIMER 7 and PERMANOVA + software (Primer-e, Auckland, New Zealand) (Anderson et al., 2008; Clarke and Gorley, 2015). All new sequences were uploaded in NCBI^4^ and are available under the submission numbers SUB10633042 (OL412293-OL412393) and SUB10648656 (OL441688-OL441757) for the SSU of *F. mosseae* and *R. irregularis*, respectively; SUB10638254 (OL435161-OL435261) for the ITS2 of *F. mosseae*; SUB10649136 (OL449343-OL449412) for the 5.8S-ITS2 of *R. irregularis*; SUB10638603 (OL422707-OL422807) and SUB10649570 (OL442685-OL442754) for the LSU of F. *mosseae* and *R. irregularis*, respectively.

## Results

### Arbuscular Mycorrhizal Fungal Root Colonization

**FIGURE 1:**
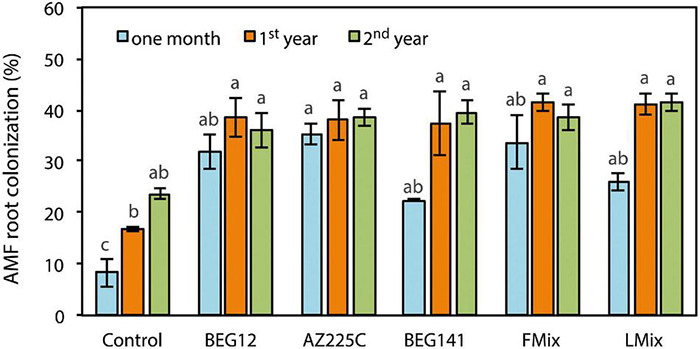
Arbuscular mycorrhizal (AM) fungal root colonization of alfalfa (*Medicago sativa* L.) as influenced by the interaction AM fungal inoculant [*Funneliformis mosseae* BEG12, *F. mosseae* AZ225C, *Rhizophagus irregularis* BEG141, foreign mixture (FMix) (BEG12, AZ225C, and BEG141), local mixture (LMix) of 14 AM fungal species belonging to five families, and mock-inoculated control] and crop stage (1 month, 1-year and 2-year). Data are mean ± SE (*n* = 3). Different letters indicate significant differences at *P* ≤ 0.001 (Supplementary Table 2).

Arbuscular mycorrhizal (AM) fungal root colonization was influenced by the interaction of AM fungal inoculant and crop stage (Supplementary Table 2). Over time and with crop aging, root colonization increased with all treatments, but the rate of increase differed among control and inoculants (Figure 1). One month after seeding, AM fungal root colonization was higher with all inoculants compared with uninoculated control, ranging from 22% for BEG141 to 34% for Fmix. However, differences among inoculants were statistically not significant. Functional root nodules were observed in all plant samples without differences among treatments (data not shown). Similarly, at the beginning of the spring growth of the first year, root colonization was higher with all inoculants compared with the uninoculated control, and the range of variation among inoculants was small (from 37% for BEG141 to 41% for Fmix). At the onset of the spring growth of the second year, root colonization increased in the uninoculated control compared with the previous year, although the difference was statistically not significant. AM fungal root colonization in the 2-year plants did not further increase with all inoculants. At this crop stage, differences in root colonization among inoculation treatments were small (36–41%) and statistically not significant. Averaged over inoculants, root colonization was 30, 39, and 39% 1 month after seeding, at the onset of the spring growth of the first year and of the second year, respectively.

### Plant Growth, Forage Yield, and N and P Content

**FIGURE 2:**
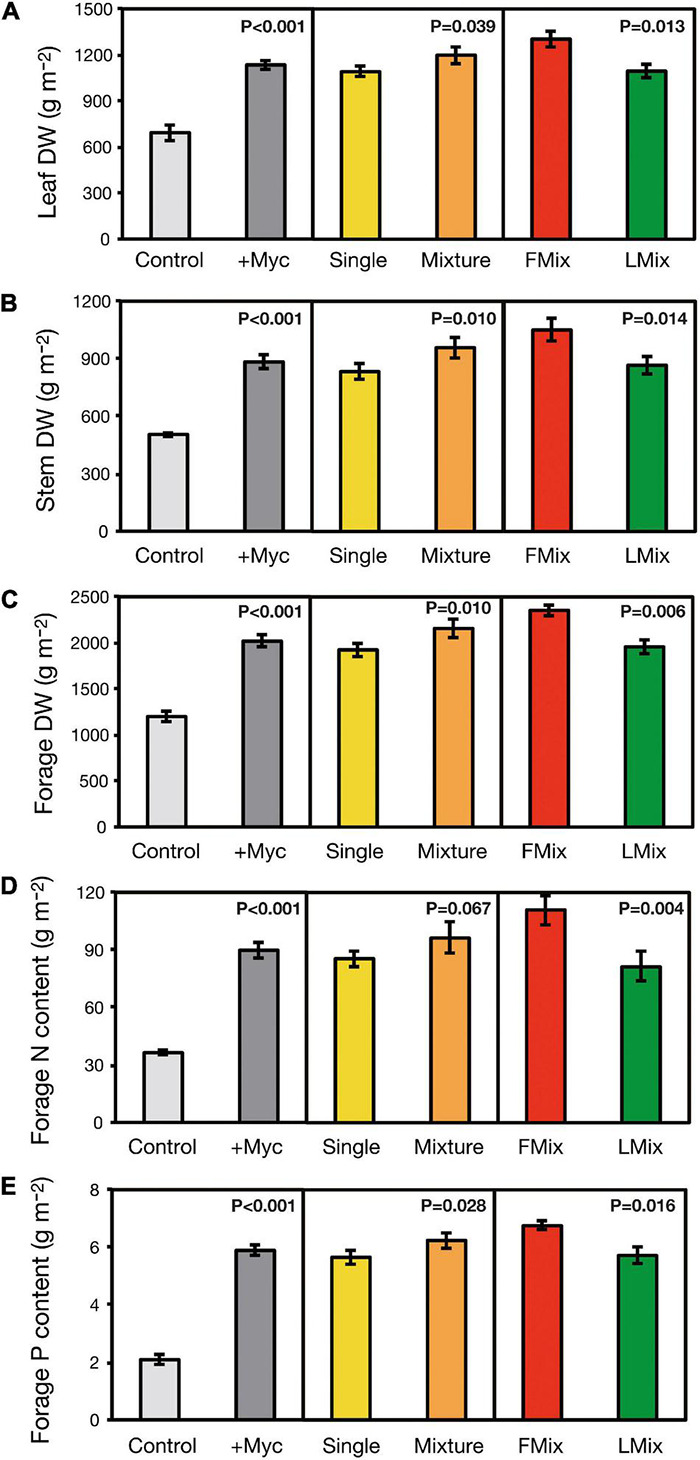
Leaf, stem, and forage dry weight **(A–C)** of alfalfa (*M. sativa*), and forage nitrogen (N) and phosphorus (P) content **(D,E)** accumulated over years and cuts as affected by AM fungal inoculation. Results of orthogonal contrasts used for discriminating the differences between means: inoculated (+ Myc) vs. control, single fungal isolates vs. mixture inoculants (Single vs. Mixture), and foreign vs. local AM fungal mixtures (FMix vs. LMix) [Inoculants: *F. mosseae* BEG12, *F. mosseae* AZ225C, *R. irregularis* BEG141, foreign mixture-FMix (BEG12, AZ225C, and BEG141), local mixture-LMix of 14 AM fungal species belonging to five families, and mock-inoculated control]. Data are mean ± SE (three plot replicates per inoculum treatment).

Leaf DW accumulated over years and cuts was increased by 64% by inoculation and was 99% higher with inoculum mixture (Fmix and Lmix) compared with single inoculants (BEG12, AZ225C, BEG141), and 19% higher with Fmix than Lmix (Figure 2A). Calculating the quantity of stem DW accumulated over years and cuts, it is evident that it was increased by 75% by inoculation, and it was 15% higher with inoculum mixture compared with single inoculants, and 21% higher with Fmix than Lmix (Figure 2B). Forage DW accumulated over years and cuts was increased by 68% by inoculation, it was 12% higher with inoculum mixtures compared with single inoculants and was 20% higher with Fmix than Lmix (Figure 2C). Forage N content accumulated over years and cuts was increased by 147% by inoculation, was unchanged when treated with inoculum mixture compared with single inoculants, and was 36% higher with Fmix than with Lmix (Figure 2D). Forage P content accumulated over years and cuts was increased by 182% by inoculation, was 10% higher with inoculum mixture compared with single inoculants, and 18% higher with Fmix than with Lmix (Figure 2E). Details about plant growth, forage yield, and N and P content across years and cuts are given in Supplementary Results and Supplementary Figures 2–4.

### Fatty Acid Profile

Arbuscular mycorrhizal (AM) fungal inoculation significantly influenced the forage FA profile of alfalfa (Supplementary Table 4). While nearly all FA examined had significant interactions between inoculation and age and between inoculation and cut, these interactions tended to be small compared with the sources of variation of inoculation. Therefore, these interactions are only reported in Supplementary Tables 5, 6, while the effect of inoculation, being of primary interest, is here illustrated. Fatty acids 18:3 (α-linolenic acid, omega-3) and 18:2 (linoleic acid, omega-6) were the predominant ones, comprising from 44 to 47% and 29 to 31%, respectively, of the total FA concentration in all treatment combinations (Supplementary Table 5). Fatty acids 16:0 and 18:1 were similar to each other in concentration, i.e., comprising from 11 to 15% and from 7 to 12% of the total FA, respectively, in all treatment combinations. Finally, FA18:0 had the smallest fraction, accounting for 2–3% of the total FA analyzed.

Arbuscular mycorrhizal (AM) fungal inoculation increased the concentration of all FA in forage compared with control, except for 18:0, and the increases were fairly similar across crop ages and cuts (Supplementary Table 5). Moreover, TFA, averaged over crop age and cut, were increased by 24% compared with uninoculated control. When significant differences occurred among inoculants, Fmix was the most effective in increasing FA concentration in alfalfa forage.

**FIGURE 3:**
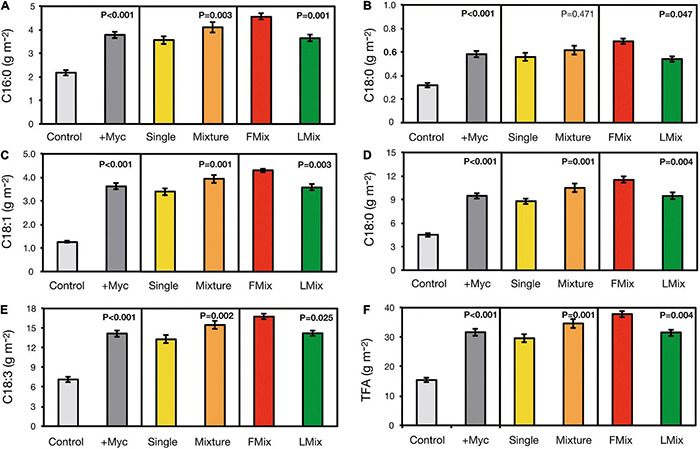
The single **(A–E)** and total fatty acid **(F)** content of alfalfa (*Medicago sativa* L.) accumulated over cuts and years as affected by AM fungal inoculation. Results of orthogonal contrasts used for discriminating the differences between means: inoculated (+ Myc) vs. control, single arbuscular mycorrhizal (AM) fungal isolates vs. mixture inoculants (Single vs. Mixture), and foreign vs. local AM fungal mixtures (FMix vs. LMix) [Inoculants: *F. mosseae* BEG12, *F. mosseae* AZ225C, *R. irregularis* BEG141, foreign mixture-FMix (BEG12, AZ225C, and BEG141), local mixture-LMix of 14 AM fungal species belonging to five families, and mock-inoculated control]. Data are mean ± SE (three plot replicates per inoculum treatment).

Fatty acids content in forage of all FA accumulated over years and cuts was increased by AM fungal inoculation, and increases ranged from 74 to 189% in 16:0 and 18:1, respectively (Figure 3). Moreover, the content of all FA, except 18:0, was higher with inoculum mixture compared with single inoculants, and the increase ranged from 10 to 19% in 18:0 and 18:2, respectively. Finally, the content of all FA was higher when plants were inoculated with Fmix than with Lmix, and the increase ranged from 18 to 28% in 18:3 and 18:0, respectively. Similarly, TFA content was increased by 105% following inoculation and was 17% greater when inoculation was performed with an inoculum mixture than with a single inoculant and 20% greater when a mixture of foreign inoculants was applied, compared with a local one.

### Molecular Discrimination Among Non-native and Native *F. mosseae* and *R. irregularis*

**FIGURE 4:**
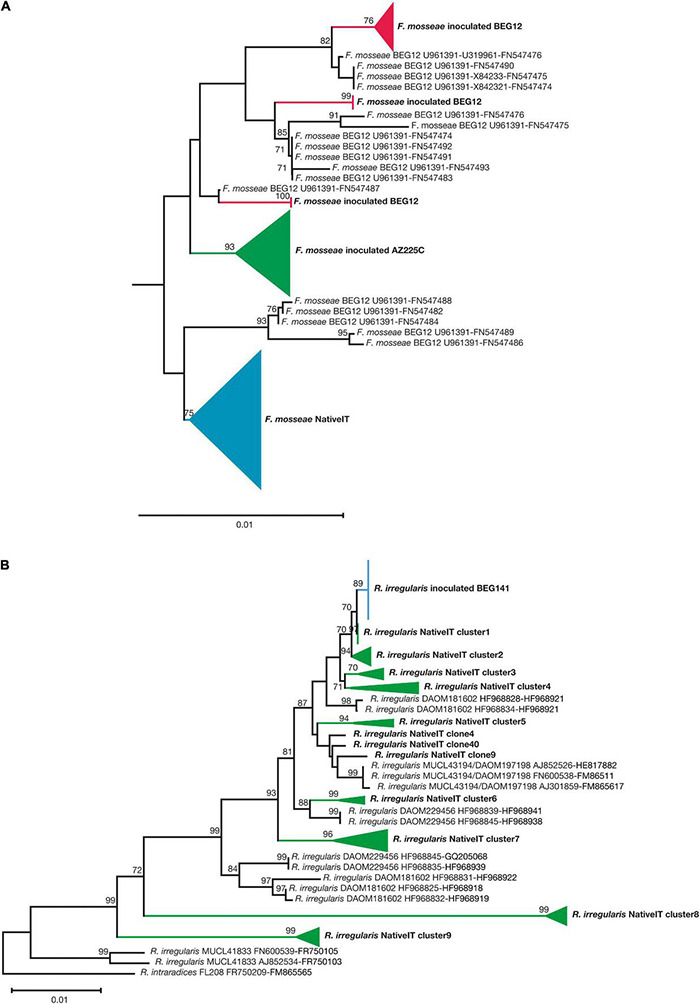
Neighbor-joining (NJ) collapsed tree of the nuclear ribosomal rDNA sequences of *F. mosseae*, originating from local strains and the non-native inoculated isolates BEG12and AZ225C **(A)**. The tree is composed of 20 and 24 newly generated sequences of BEG12 and AZ225C, respectively, 39 native *F. mosseae* sequences, plus 17 sequences of BEG12 as reference. NJ collapsed tree of the nuclear ribosomal rDNA sequences of *R. irregularis*, originating from local strains and the non-native inoculated isolates BEG141 **(B)**. The tree is composed of 17 newly generated sequences of BEG141 and 40 native *R. irregularis* sequences, plus 14 sequences of *R. irregularis* isolates as reference (DAOM181602, MUCL43194/DAOM197198, DAOM229456, and MUCL41833) The NJ *F. mosseae* tree is based on concatenated sequences of the small subunit ribosomal RNA gene (SSU; ca. 550 bp), the internal transcribed spacer 2 (ITS2; ca. 165 bp) and the large subunit ribosomal RNA gene (LSU; ca. 400 bp). The NJ *R. irregularis* tree is based on concatenated sequences of the small subunit ribosomal RNA gene SSU (ca. 720 bp), 5.8S, and ITS2 (ca. 430 bp), and LSU (ca. 365 bp). Bootstrap values (based on 1,000 replicates) are shown at the nodes. The scale bar indicates substitutions per site. Clades formed by sequences of native and inoculated *F. mosseae* and *R. irregularis* strains are shown by colored branches and triangles. The concatenated sequences AJ245637-AJ239122-AJ5110241 of the isolate BEG11 of *Funneliformis geosporum* were used as an outgroup to root the *F. mosseae* tree, while the concatenated sequences FR750209-FM865565 of the isolate of *R. intraradices* FL208 were used as an outgroup to root the *R. irregularis* tree. The *F. mosseae* tree is outgroup-truncated. The newly generated sequences are highlighted in boldface, and their accession numbers are indicated in Supplementary Figure 3.

The c. 2,200-bp-long central stretch of the nuclear rDNA cistron, obtained using the PCR primers NS31 and LSUGlom1, provided sufficient phylogenetic resolution to discriminate the inoculated isolates from native strains of *F. mosseae* and *R. irregularis*. However, similarly to Pellegrino et al. (2012), in the case of *F. mosseae* BEG12 and AZ225C, the discrimination was possible using the concatenated sequences of the variable 3′ end of the SSU rRNA gene (ca. 550 bp), ITS2 (ca. 165 bp), and the variable 5′ end of the LSU (400) rRNA gene (Figure 4A and Supplementary Figure 5). In the case of *R. irregularis* BEG141, the discrimination was possible using longer concatenated sequences of the variable 3′ end of the SSU rRNA gene (ca. 720), the 5.8S and ITS2 (ca. 430 bp), and LSU (ca. 365 bp) (Figure 4B and Supplementary Figure 6). The sequences (obtained from spores) of the inoculated *F. mosseae* BEG12 and AZ225C, and *R. irregularis* BEG141 clustered distinctly from those of the native strains of both AM fungal species, amplified from the roots of Sudan grass grown in pots for the production of the Lmix inoculum (Figure 4 and Supplementary Figures 5, 6). The partial sequences of the nuclear rDNA cistron of the *F. mosseae* BEG12 isolate clustered into two phylogenetic subclusters, whereas those of *F. mosseae* AZ225C and *R. irregularis* BEG141 both grouped into only one cluster.

Reference sequences of *F. mosseae* BEG12 from NCBI clustered together with the sequences two phylogenetic subclusters of the inoculated *F. mosseae* BEG12 maintained in cultivation since 2010 at the AM fungal bank of the Institute of Life Science (Scuola Superiore Sant’Anna, Pisa, Italy) (Figure 4A and Supplementary Figure 5). Since no reference sequences of SSU and ITS2 were available for *R. irregularis*, BEG141 and only three sequences available for LSU were included in the alignment as input for the tree building of four different isolates of *R. irregularis* and one of *R. intraradices* (Figure 4B and Supplementary Figure 6). The sequences of *R. irregularis* MUCL43194/DAOM197198 grouped into a unique, well-supported cluster, as well as the sequences of *R. irregularis* MUCL41833. Conversely, the sequences of *R. irregularis* DAOM181602 were grouped into two distinct clusters, as well as those of DAOM229456. The sequences of the local strain of *F. mosseae* were grouped into one cluster, whereas those of *R. irregularis* were more divergent and grouped into nine distinct clusters (Figure 4 and Supplementary Figures 5, 6).

### Tracing Non-native and Native Strains of *F. mosseae* and *R. irregularis* in the Field

**FIGURE 5:**
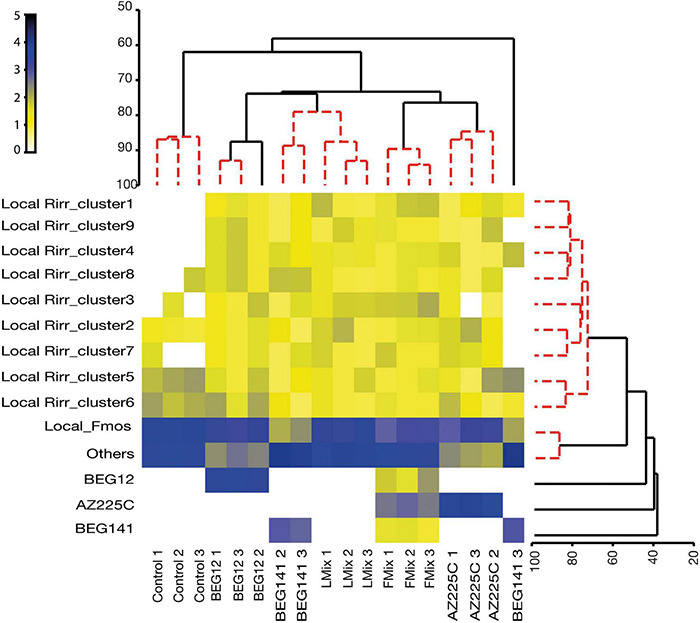
Dendrogram of Similarity Profile (SIMPROF) cluster analysis (horizontal dendrogram) grouping the different samples collected at the beginning of spring growth (roots of 2-year alfalfa, *M. sativa*) [three fields replicate plots treated by mock-inoculated control (Control), *F. mosseae* isolates BEG12 and AZ225C, *R. irregularis* isolate BEG141, foreign mixture-FMix (BEG12, AZ225C, and BEG141), local mixture-LMix of 14 AM fungal species belonging to five families] into clusters based on their similarity/homogeneity of AM fungal intraradical communities [relative abundances of phylotypes of non-native/inoculated *F. mosseae* BEG12 and AZ225C, non-native/inoculated *R. irregularis* BEG141, native *F. mosseae* (Local Fmos) and native *R. irregularis* (Local Rirr_cluster1-9) and other Glomeromycota taxa (others)]. Vertical dendrogram groups the different phylotypes based on their similarity of occurrence. Red clusters are supported by the SIMPROF analysis. Relative abundance input data were log(X + 1) transformed and Bray-Curtis similarity was calculated and shown as a shaded plot.

Using the validated molecular markers, foreign and local strains of *F. mosseae* and *R. irregularis* were successfully traced within the roots of the polyannual forage crop alfalfa 2 years following inoculation. However, AZ225C was the most persistent both as single inoculum or mixture, followed by BEG12 and BEG141 (Supplementary Table 7). This is also evidenced by the cluster analysis and the associated shade plot based on the AM fungal community found in the inoculated and control plots (Figure 5). Control replicates clustered together and separately from the inoculated plots, which were separated according to inoculation treatment, except for BEG141. Overall, local *F. mosseae* were similarly abundant to the other Glomeromycota taxa, but they were differently retrieved in the treated plots. In the BEG12 and AZ225C plots over 60% of the retrieved sequences were assigned to *F. mosseae* either inoculated or local, whereas in the BEG141 plots local *F. mosseae* accounted only for 7.4% (Supplementary Table 7). Comparing control and Lmix, local *F. mosseae* and other Glomeromycota taxa accounted for 44 and 37% of the total retrieved taxa in the control plots, while they accounted for 39 and 45%, respectively, in the Lmix plots. Finally, clusters 1–9 of local *R. irregularis* formed a unique group across the treated replicates, although clusters 5 and 6 were more abundant in the control, BEG12, and BEG141 plots.

These results are also supported by the NJ trees built focusing on *F. mosseae* and *R. irregularis* and by the relative abundance pie charts based on inoculum treatments (Supplementary Figures 7, 8). In detail, the NJ tree of *F. mosseae* clearly showed that the sequences retrieved in the alfalfa roots collected from the plots inoculated by BEG12, AZ225C, and Fmix clustered into three groups, phylogenetically affiliated to BEG12, AZ225C, and the local sequences of *F. mosseae*. Among these, 86% of the sequences affiliated to BEG12 were retrieved in the plots originally inoculated with this isolate, while the remaining were retrieved in the Fmix plots (14%) and no sequences were found in the other treatments. Similarly, 81% of the sequences affiliated to AZ225C were retrieved in the plots inoculated with AZ225C and 19% in the Fmix plots. Finally, the sequences phylogenetically affiliated to the native *F. mosseae* were retrieved in all the inoculated plots but were more abundant in the control plots, and their occurrence decreased progressively from Lmix (30%) to BEG141 (7%) (Supplementary Figure 7). As regard *R. irregularis*, the NJ tree showed that the sequences retrieved in the alfalfa roots collected from the treated plots were grouped into 10 clusters (Supplementary Figure 8), one cluster composed of sequences phylogenetically affiliated to BEG141 and the others composed by the sequences affiliated to the nine clusters of local *R. irregularis* strains initially found (Figure 4B). About 88% of the sequences affiliated to BEG141 were retrieved in the plots originally inoculated with this isolate, while the remaining were retrieved in the Fmix plots (12%) and no sequences were found in the other treatments. Regarding local *R. irregularis* clusters, the relative abundance of clusters 1, 4, and 6 differed among treatments, while the abundances of the others did not change (Supplementary Figure 8).

### Exploring the Relationship Between the Components of Arbuscular Mycorrhizal Fungal Communities in Alfalfa Roots and Plant Traits

**FIGURE 6:**
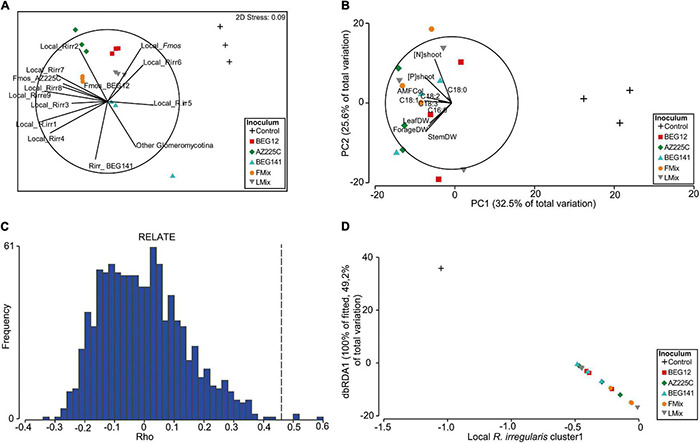
Non-metric multidimensional scaling analysis (nMDS) plot based on the Bray-Curtis similarity matrix calculated on the fourth-root AM fungal relative abundances of phylotypes retrieved within the roots of inoculated and not-inoculated 2-year alfalfa **(A)**. A principal component analysis (PCA) plot was performed on the Euclidian distance matrix calculated on the square root of the plant traits (plant growth, N and P concentration of forage, and fatty acid composition) **(B)**. RELATE analysis based on Spearman rank and 999 permutations for testing the significance of the relationship between the two matrices **(C)**. Distance-based redundancy analysis plot used to visualize the first and second axes of the Distance-based linear method (DistLM) analysis applied to measure the significance and the variance explained by the best descriptor **(D)**.

In the nMDS plot, representing the AM fungal communities within the roots of the 2-year alfalfa, the inoculum treatments were well separated along a first and second axis (Figure 6A), and the nMDS stress (0.09) supported this representation of the dissimilarity among samples. Moreover, the PCA, based on the corresponding plant traits, well separated the non-inoculated plots from the inoculated ones along the first axis, as well as the different inoculants along the second axis (Figure 6B). Overall, local *R. irregularis* clusters 1–4, and 7–9, and *F. mosseae* AZ225C and BEG12 were positively and strongly correlated with all plant traits. The relationship of the two matrices was also shown to be significant by the RELATE analysis, for which the ρ was equal to 0.46 and the significance level of sample statistic after 999 permutations were equal to 0.4% (Figure 6C). Nevertheless, the BEST analysis allowed us to highlight that together local *R. irregularis* cluster1 and cluster6 were the best predictors of the plant traits (correlation:0.656). Finally, although the marginal test of the DistLM analysis highlighted that many phylotypes were significantly correlated with the plant traits and explained a relevant part of the total variation (Supplementary Table 8), the local *R. irregularis* cluster1 alone was sufficient to describe the majority of the diversity inside the plant traits (49.2% of the total variance explained; *P* = 0.003). This is also detectable from the plot of the dbRDA of the DistLM axes (Figure 6D) showing that the increase in the abundance of the local *R. irregularis* cluster1 well explains the distribution of the samples in the multivariate space based on plant traits (Figure 6B).

## Discussion

### Arbuscular Mycorrhizal Fungal Inoculation Enhances Alfalfa Yield and Fatty Acid Concentration in Forage

Overall, AM fungal field inoculation enhanced crop yield and FA concentration in both years of cultivation. AM fungal root colonization was greatly increased by inoculation and the effect was the highest at the beginning of crop growth. Indeed, already 1 month following inoculation, all inoculants reached the saturation (ca. 40%) within the roots of alfalfa, compared with control having less than 10% of colonization. This suggests a high infectivity of the AM fungal inoculants under field conditions. This is in agreement with the root colonization pattern reported in a previous study on alfalfa field-inoculated with two single isolates of *F. mosseae* (IMA1 = BEG12 and AZ225C) (Pellegrino et al., 2012). In the study of Pellegrino et al. (2012), already 3 months after crop emergence, AM fungal colonization in the inoculated plants reached 30%, while in the control plots were low (3%), and the rate of colonization did not change with crop aging, supporting the high infectivity of the inoculants on alfalfa and a saturation level similar to the one in our research. Our data evidenced that native AMF occurring into control plots were less infective, because up to 2 years from crop seeding their presence in plant roots was still lower compared with inoculated plots. Thus, these results support the high infectivity (54%–80%) found in a pot experiment with a 4-month-old alfalfa inoculated by *F. mosseae* IMA1 and AZ225C, and by *R. irregularis* IMA6 (= BEG141) (Avio et al., 2006). Other studies carried in pots with isolates of *F. mosseae* and *Rhizophagus intraradices*, reported in 8-month-old alfalfa plants root colonization up to 60% (Wang et al., 2020; Li et al., 2021). Thus, our results confirm the high mycotrophic behavior of this important forage crop previously described in controlled conditions.

In the first year of growth, the higher AM fungal root colonization detected in inoculated alfalfa compared with uninoculated controls was associated with a general improvement of plant growth, due to an increase of the growth of stems and leaves and consequently of forage. However, this fungal trait could not fully explain the variability in plant growth, since inoculants colonizing the roots of alfalfa at a similar rate differently influenced plant growth. In this regard, Lekberg and Koide (2005) found a significant but weak relationship between a change in mycorrhizal colonization and a change in yield. Therefore, while an increase in mycorrhizal colonization appears to be associated with an increase in yield, the mycorrhizal colonization cannot be the sole predictor of an increase in yield, and the characterization of the AM fungal components of the root community assemblages became essential for dissecting functionality (Vályi et al., 2016). Indeed, AMF taxa can greatly differ in the spread of external mycelium or hyphae architecture, thus affecting yield and nutrient uptake (Munkvold et al., 2004; Avio et al., 2006).

The positive effect of field AM fungal inoculation on N and P concentration in forage irrespective of inoculants is consistent with the results on Egyptian clover (*Trifolium alexandrinum* L.) and alfalfa (Pellegrino et al., 2011, 2012). A generalized positive effect of AM fungal inoculation on plant P concentration was reported by Lekberg and Koide (2005) taking into consideration 25 field trials. About the effect of AM fungal inoculation on plant N concentration, while a generalized increase was reported by Delavaux et al. (2017) looking only to greenhouse trials, the effect in the field is less clear and poorly studied (Pellegrino et al., 2015a). However, in Pellegrino et al. (2011) a strong variability among AM fungal inoculants was reported on forage nutrient concentrations in pots where no other AMF occur. This suggests that the interactions of AM fungal inoculants with the native AM fungal community are likely to be responsible for the lack of differences in fungal physiological traits (van der Heijden and Scheublin, 2007). In addition, in the field, the outcome of the inoculation is influenced also by the disturbance history (i.e., reduced disturbance and shorter fallow) (Lekberg and Koide, 2005), and/or by soil nutrient availability (i.e., N and P) and soil pH (Han et al., 2020).

So far, a unique study in a field-grown crop analyzed the effect of AMF on fatty acid content and composition (Igiehon et al., 2021). In contrast with our results highlighting an increase in the amount of fatty acids, Igiehon et al. (2021) found a lower amount of fatty acids in seeds of soybean when plants were inoculated with AMF and no changes of fatty acids when soybean was inoculated with AMF plus strain of *Rhizobium* sp. However, similar to our results, the composition of polyunsaturated FA (PUFA), monounsaturated FA (MUFA), and saturated FA (SFA) was greatly modified by AMF and *Rhizobium* in comparison with the uninoculated control. Moreover, drought stress was shown to enhance the fatty acid contents in seeds of some inoculated soybean plants through upregulation and downregulation of specific fatty acids (Hou et al., 2006; Igiehon et al., 2021). Under water-deficit conditions, plants colonized by mycorrhizal fungi have better growth than those that are not, and the response is more evident in perennial plants (Jayne and Quigley, 2014). Therefore, our results largely confirm the hypothesis that AM fungal inoculation supports plant growth and total fatty acid content in drought conditions, likely to occur in the area where our trial was run. A higher concentration of oleic acid (C18:1), linoleic acids (C18:2), and α-linolenic acid (C18:3), we found in all inoculated alfalfa plots, is a desirable quality in forage for animal nutrition, since these components are associated with several health and nutritional benefits (Radović et al., 2009; González-Fernández et al., 2020). The fatty acid auxotrophy of AMF is supported by recent studies showing that lipids synthesized by the host plants (e.g., palmitic, oleic, and myristic acids) are transferred to the fungi, lacking genes encoding fatty acid synthases (Stumpe et al., 2005; Trépanier et al., 2005; Sugiura et al., 2020). However, our results suggested that AMF, in addition to using fatty acids as C and energy source, can stimulate their synthesis in the aboveground part of the plant. This is in line with recent results reporting that AMF is involved in the stimulation of the synthesis of secondary metabolites plant (Baslam and Goicoechea, 2012; Zouari et al., 2014; Kaur and Suseela, 2020). Moreover, the use of AM fungi in pasture management could provide benefits for animals, derived products, and humans. In this regard, cows grazing in AM fungal inoculated pasture produced high-quality milk (Masoero et al., 2021).

### Multiple Arbuscular Mycorrhizal Fungal Inoculants Act in Synergy to Promote Alfalfa Yield and Fatty Acids Concentration in Forage

In this study, mixed AM fungal inocula performed better than single inocula both in terms of plant growth, P uptake, and FA content and composition. However, no difference in AM fungal root colonization was reported. Several studies carried out in pot conditions with sterilized substrate showed that AM fungal mixed inocula promoted markedly plant performance (e.g., Edathil et al., 1996; Maherali and Klironomos, 2007; Thonar et al., 2014; Martignoni et al., 2021), whereas the few studies performed in the field do not allow to reach conclusive assessments (e.g., Powell, 1977, 1979; Clarke and Mosse, 1981; Pellegrino et al., 2011; Pellegrino and Bedini, 2014). Recently, some meta-analyses synthesized the literature on this topic, but merging the data obtained from lab experiments with the few obtained from the field (e.g., Rúa et al., 2016; Yang et al., 2017; Zhang et al., 2019). Significant differences were found in cereal yield (i.e., wheat, rice, and corn) if mixed inocula were compared with single inocula and substrate sterilization was not applied, whereas no differences were found if the substrate sterilization was applied with or without application of a microbial supplement (Zhang et al., 2019). These results may derive from complementarity among AM fungal species, which can provide a greater crop benefit (Koide, 2000; Thonar et al., 2014). A mixed and more complex inoculum can be more efficient across a range of soil parameters, climatic conditions, and host plants (species or even varieties/genotypes) increasing plant nutrient uptake, decreasing nutrient losses, and improving soil aggregation. The greater efficiency of mixed inocula can be the result of a large variability among AM fungal taxa in foraging strategies and extraradical mycelium development (spread of the mycelium, hyphal architecture, or anastomosis frequency). This variability may enhance the ability of the inoculum to adapt and thus be more efficient and/or more persistent in many environments. Indeed, the structure of the AM fungal extraradical mycelium (ERM) that is largely variable among AM fungal species or even isolates among species was linked to specific functions (Munkvold et al., 2004; Avio et al., 2006; Thonar et al., 2011; Treseder et al., 2018). For example, a high positive correlation was highlighted between ERM interconnectedness (anastomosis frequency and number) and plant growth variables (total shoot biomass), or between anastomosis frequency and root P content (Avio et al., 2006). Moreover, a crop genotype inoculated with an AM fungal mixture may have a great probability to find among the AMF components the taxa with a higher host preference and a life history strategy adapted to local agricultural soil. In this regard, Wagg et al. (2015) using a model plant-mycorrhizal system, suggested that the fungal composition rather than the diversity of species may be more important in determining how plant species function within a community.

### Multiple Arbuscular Mycorrhizal Fungal Inoculants Composed of Foreign Arbuscular Mycorrhizal Fungi Provide Greater Benefits to Alfalfa Compared to a Mixture of Local Arbuscular Mycorrhizal Fungi

Foreign isolates combined in the mixture performed better than the local-isolate mixture composed of 14 AM fungal species belonging to seven genera and five families. This is in agreement with the results recorded in the field by Pellegrino et al. (2011) On the contrary, Pellegrino and Bedini (2014) reported that the locally sourced AM fungal mixed inoculum was more effective than a foreign mixture inoculum. The greater effectiveness of foreign mixtures is in accordance with the results of the meta-analysis of Rúa et al. (2016) concluding that the geographic origin of plants relative to the origin of AMF and soil is important for describing the effect of inoculation on plant biomass. Indeed, if plants and soil are sympatric (i.e., locally sourced), but allopatric to the fungus (i.e., foreign origin), the positive effect of inoculation is greater than when all three components are allopatric. In agreement, Klironomos (2003) reported positive responses using local plants and fungi, and parasitic or no responses using foreign plant or fungal genotypes. Kiers et al. (2011) explained this concept by the bidirectionality of mutualism and the reward for individuals showing the best rate of exchange. Indeed, plants and fungi are able to detect variation in the resources supplied by their partners, allowing them to adjust their own resource allocation accordingly. In addition to this concept, the taxonomic resolution of the AM fungal diversity in the inocula is the main factor of plant functionality, as the functional benefit was found at AM fungal family level, and not at species scale (Yang et al., 2017). Thus, an inoculum composed of many AM fungal families would allow maintaining a full complement of ecosystem functions, rather than an inoculum composed of many species belonging to the same o few families. Therefore, our hypothesis of greater host benefits from local AM fungal isolates than foreign isolates has to be rejected, probably due to the legacy of the agricultural practices (tillage, fertilization, pesticides, etc.) potentially selecting for less advantageous AM fungal strains. Finally, we would like to highlight, as previously mentioned, that in this study inoculants were composed of spores, roots, and soil (crude inoculum). Thus, during the process of production of the different AM fungal isolates and mixtures, AMF may have had socialized differently with the microbial community. This means that, although a significant microbial filtrate was applied at the start of the process of production to minimize microbial differences, at the end of the production phase the variabilities in crop response might be due not only to the AM fungal species identity but also to differences in microbial associated groups. Indeed, from the mycorrhizosphere and the spores of different AM fungal isolates many different bacterial taxa were detected (Bharadwaj et al., 2008). The multiple services provided by AMF were shown to be the result of the synergistic activity of the diverse bacterial communities living in the mycorrhizosphere, strictly associated with the spores and with the extraradical mycelium, which played many plant growth-promoting roles (e.g., nitrogen fixation, P solubilization, production of indole acetic acid, siderophores, and antibiotics) (Frey-Klett et al., 2007).

### Molecular Discrimination of Foreign Inoculants and Native *F. mosseae* and *R. irregularis*

In this work, consistently with Pellegrino et al. (2012), the phylogenetic information contained in the polymorphic 3′ end of the nuclear SSU rRNA gene, ITS2, and the 5′ end of the LSU rRNA gene were sufficient for discriminating among foreign inoculants and local strains of *F. mosseae*. Moreover, the phylogenetic information contained in the 5.8S in addition to that contained in the partial SSU, complete ITS2, and partial LSU, allowed also to discriminate foreign inoculants and local isolates of *R. irregularis*. Thus, in our field site, these regions gave sufficient variation to reach a good resolution among closely related AM fungal isolates for both AM fungal species. Molecular discrimination of foreign inoculants might have been facilitated by the fact that both *F. mosseae* isolates BEG12 and AZ225C, and *R. irregularis* BEG141 have a distant origin. Indeed, *F. mosseae* isolates BEG12 and AZ225C are originated from United Kingdom and Arizona (United States), and *R. irregularis* BEG141 from France. Despite the fact that a high rDNA sequence polymorphism was proved within individuals (e.g., Hijri et al., 1999; Lanfranco et al., 1999; Jansa et al., 2002; Thiéry et al., 2012), other researchers (Krüger et al., 2009, 2012; Stockinger et al., 2010), similarly to our findings, proved that the combination of the highly polymorphic ITS region with variable SSU and LSU regions can be used to achieve phylogenetic species- and strain-level resolution in AMF. In this regard, Farmer et al. (2007) could discriminate isolates of *R. irregularis* BEG141 and *C. etunicatum* BEG168, using only the variable domains of the LSU of ribosomal RNA gene, whereas more recent studies proved that other molecular markers (i.e., mtLSU, RPB1, and mt cox3-rnl intergenic mtDNA region) are even more efficient in the discrimination of foreign AM fungal isolates in the field (Sýkorová et al., 2012; Buysens et al., 2017; Kokkoris et al., 2019; Thioye et al., 2019). Very recently, to overcome the difficulties of the lack of consensus barcoding region for the determination of AM fungal taxa, Kolaříková et al. (2021) developed a fine and promising approach to sequence an AM fungal marker within the ribosome-encoding operon (rDNA) which covers all the three widely applied variable molecular markers (SSU, ITS, and LSU). In detail, they amplified a ca. 2.5 kb of the rDNA, spanning the majority of the SSU gene, the complete ITS region, and a part of LSU rRNA gene, and sequenced the PCR products using a PacBio platform and the Single-Molecule Real-Time sequencing. This approach proved to be able to obtain a robust phylogenetic assignment of several Glomeromycota lineages occurring in complex AM fungal assemblages, could overcome the site- and inoculation-specificity of the molecular approach we proposed.

### Persistence of Inoculated Arbuscular Mycorrhizal Fungi in the Field and Effects on Native Arbuscular Mycorrhizal Fungi

Using the validated molecular approach, foreign strains of *F. mosseae* and *R. irregularis* were found to persist in the roots of the poliannual forage crop alfalfa 2 years following inoculation with single-foreign isolate inoculum or the mixture of those isolates. Similarly, in potatoes, the inoculated *R. irregularis* BEG141 and *R. etunicatum* BEG168 were found to persist in roots 6 weeks following inoculation (Farmer et al., 2007). In this study, a nested PCR using taxon-specific primers, targeting the variables domains of the LSU of ribosomal RNA gene, and a cloning-sequencing approach was applied. Moreover, using other molecular markers, such as the mtLSU (cloning and sequencing approach) and a fragment of RPB1 (qPCR approach), Sýkorová et al. (2012) and Thioye et al. (2019) successfully traced two haplotypes of *R. irregularis* BEG140 in roots of field-inoculated *Phalaris arundinacea* and *R. irregularis* IR27 in roots of jujube (*Ziziphus mauritiana*), respectively. *Rhizophagus irregularis* BEG140 was reported to survive and proliferate up to 3 years in the field and was found more often in the inoculated than in control plots (Sýkorová et al., 2012), while *R. irregularis* IR27 was reported to persist in jujube tree roots up to 18 months. More recently, Buysens et al. (2017), using a qPCR approach and amplifying the mtLSU RNA gene, performed for the first time the absolute quantification in the field of the isolate *R. irregularis* MUCL 41833, and found a significant correlation between mtLSU_MUCL41833 and total, arbuscular and vesicular colonization of roots of three varieties of potatoes at three growing stages (i.e., flowering, before defoliation and harvest). Finally, Kokkoris et al. (2019) successfully traced the *R. irregularis* DAOM 197198 using specific primers, targeting the mt cox3-rnl intergenic mtDNA region, and a probe in a droplet digital PCR. The inoculated isolate was found in all the plots (inoculated and non-inoculated) and its abundance increased over time, supporting the hypothesis of the spreading of the isolate beyond inoculated plots. Conversely, Berruti et al. (2017), using a next-generation sequencing-based on the amplification of only ca. 420 bp of the 18S V3-V4 hypervariable domains (SSU), found a lack of field persistence of the AM fungal components of microbial mixed inoculum in maize at the V8-9 stage. Therefore, long-term inoculation trials are needed for monitoring the evolution of the AM fungal native community and to track the persistence of the inoculated AMF.

In our study, among the foreign-inoculated AM fungal isolates, *F. mosseae* AZ225C was the most persistent both as single inoculum or mixture, followed by BEG12 and BEG141. This is in agreement with a previous study (Pellegrino et al., 2012), where at 2 years post-inoculation, the isolate AZ225C was more persistent in roots of *M. sativa* with respect to IMA1, although it declines from the establishment (i.e., 3 months following plant emergence; from 100 to 16%). In the previous study (Pellegrino et al., 2012), IMA1, despite still stimulating crop performance, did not persist after 2 years. This inconsistency proves the high site-specificity of the outcome of the inoculation related to climate, texture, and chemical properties of soil (Ciccolini et al., 2015; Pellegrino et al., 2015a,b, 2020a), or the genetic variability in AM fungal compatibility with different genotypes of the same plant species (*M. sativa* L. var. Giulia vs. cv. Messe) (Pellegrino et al., 2012). In this regard, a large variability in compatibility was proved for AMF in *Triticum turgidum* (Singh et al., 2012), and in *Triticum aestivum* L. (Pellegrino et al., 2020a).

Comparing mock-inoculated control and LMix, while the abundance of the local *F. mosseae* cluster was reduced, the abundance of the native *R. irregularis* clusters and other Glomeromycota taxa was promoted. The change of assemblages with LMix in comparison with those observed with single foreign inoculants of *F. mosseae* BEG12 and AZ225 (i.e., a strong decrease in the abundance of other Glomeromycota), supports the fact that inoculation with local AM fungal mixture or with the less persistent *R. irregularis* BEG141 determines a less environmentally impacting shift of the local fungal community. This is also associated with similarities in the outcome of the symbiosis in terms of plant performance (i.e., plant growth and nutrient and FA content).

The fact that sequences phylogenetically affiliated to the local *F. mosseae* were retrieved in all the inoculated plots, but were more abundant in the control plots, support the high ubiquity of this species (generalist) across a wide range of agro-climatic conditions and geographically areas (Öpik et al., 2006). Finally, no sequences similar to those of BEG12, AZ225C, and BEG141 were found in the roots of mock-inoculated alfalfa (controls) or the roots of alfalfa inoculated with LMix. This could support the absence of spreading of the inoculated AM fungal isolates, at least up to 2 years following inoculation and in such specific management, and in such a no-tilled polyannual forage. However, we cannot exclude a low fungal abundance as well as an insufficient sampling effort due to the used cloning and Sanger sequencing approach. Finally, since we inoculated crude inocula and the method of propagation cannot fully eliminate differences in microbial communities, we could not exclude that the outcome of field persistence can be due not only to the identities of inoculated AMF but also to the associated microbes that might affect the establishment/persistence, with consequences in the soil/rhizospheric microbial communities.

### Components of Arbuscular Mycorrhizal Fungal Communities in Alfalfa Roots Best Explain Plant Traits

Unexpectedly, although a high diversity in the composition of AM fungal assemblages was found among inoculated treatments, small differences were found in plant traits, such as growth and nutrient uptake, with the exception of FA. Total fatty acids (TFA) were increased with inoculum mixtures compared with single inoculants, while the content of the majority of FA was higher when plants were inoculated with FMix than with LMix. However, by relating the AM fungal assemblages within the roots of the 2-year alfalfa with the corresponding plant traits, the only change in abundance of the local *R. irregularis* cluster1 induced by all inoculation treatments was sufficient to describe the majority of the diversity inside the overall studied plant traits. Thus, we can speculate that the main determinant of the improved host benefit across the inoculation treatments is related to the stimulation of the proliferation of a single-taxa rather than to the increase in abundance of the inoculated isolates. Overall, these results suggest an environmental-driven selection for highly efficient AMF and support the use of native mixture inoculants having a lower risk of including invasive isolates (Hart et al., 2017).

## Conclusion

The data presented in this study demonstrated that: (i) field application of AM fungal inocula improved alfalfa yield, nutrient and fatty acid concentration up to 2 years post-inoculation and enhanced AM fungal abundance in roots; (ii) multiple AM fungal inoculants can act in synergy to achieve the effects on crop yield and quality; (iii) a mixture of foreign AM fungal isolates provided greater benefits compared to local consortia; (iv) foreign strains of *F. mosseae* and *R. irregularis* persisted in the roots of the polyannual crop 2 years following inoculation either as single inoculum or as component of the mixture; (v) among inoculants, *F. mosseae* BEG12 and AZ225C and the foreign mixture exerted an higher environmental impact on the local AM fungal community compared with the local AM fungal mixture and *R. irregularis* BEG141; (vi) the stimulation of the proliferation of a single-taxa, such as the local *R. irregularis* cluster1, induced by all inoculation treatments was the main determinant of the improved host benefits, suggesting an environmental-driven selection for a highly efficient AM fungal strain. The results on crop productivity and quality as well as on-field persistence of inoculated AMF support the use of mixtures of foreign AM fungal isolates. On the other hand, taking into consideration the effects on the native AMF, the local AM fungal mixture shows a lower impact. These results pave the way for extending the study on the effect of exotic and native mixtures for the production of high-quality forage for the animal diet, allowing to assess the fatty acid profile in milk or meat, and derived products, and finally to evaluate AM fungal benefits on human health.

## Data Availability Statement

The datasets presented in this study can be found in online repositories. The names of the repository/repositories and accession number(s) can be found in the article/Supplementary Material.

## Author Contributions

EP conceived and designed the study and collected and analyzed the samples. EP and LE analyzed the data. EP, LE, and MN discussed the results. EP and LE wrote the manuscript. All authors reviewed and approved the manuscript before its submission.

## Conflict of Interest

The authors declare that the research was conducted in the absence of any commercial or financial relationships that could be construed as a potential conflict of interest.

## Publisher’s Note

All claims expressed in this article are solely those of the authors and do not necessarily represent those of their affiliated organizations, or those of the publisher, the editors and the reviewers. Any product that may be evaluated in this article, or claim that may be made by its manufacturer, is not guaranteed or endorsed by the publisher.

## Footnotes

1
https://mafft.cbrc.jp/alignment/server/large.html

2
http://doua.prabi.fr/software/seaview

3
https://www.megasoftware.net

4
https://submit.ncbi.nlm.nih.gov
