# Supplementary material for: Multiple Arbuscular Mycorrhizal Fungal Consortia Enhance Yield and Fatty Acids of Medicago sativa: A Two-Year Field Study on Agronomic Traits and Tracing of Fungal Persistence
Source: Front Plant Sci. 2022 Feb 14;13:814401. doi: 10.3389/fpls.2022.814401 (PMC8882620; doi:10.3389/fpls.2022.814401)
Supplement: Supplementary file 1 [file Data_Sheet_1.docx]

Supplementary Material

# Supplementary Figures and Tables

## Supplementary Tables

| **Table S1**\| Geographic origin and inoculum source of arbuscular mycorrhizal fungal isolates used in the present work. | | | | |
| --- | --- | --- | --- | --- |
| Fungal species | Isolate code | Geographical origin | Collector | Original inoculum supplier |
| *Funneliformis mosseae* | BEG12 | Kent, UK | B. Mosse | BEG, Djion France |
| *Funneliformis mosseae* | AZ225C | Arizona, USA | J. C. Stutz | INVAM, Morgantown, W. Va. |
| *Rhizophagus irregularis* | BEG141 | France | V. Gianinazzi-Pearson | BEG, Djion France |
| ^a^BEG, Bank of European Glomales; ^b^INVAM, International Culture Collection of Arbuscular and Vesicular-Arbuscular Mycorrhizal Fungi. | | | | |

| **TABLE S2 \|** *P*-values of two-way ANOVAs evaluating the effect of arbuscular mycorrhizal (AM) fungal inoculant (Inoc) and crop stage (Stage) on AM fungal root colonization of alfalfa (*Medicago sativa* L.). | |
| --- | --- |
| Treatment^a^ | AM fungal root colonization |
| Inoc | **<0.001**^b^ |
| Stage | **<0.001** |
| Inoc x Stage | **<0.001** |
| *^a^General mixed model including Inoc [*Funneformis mosseae *BEG12,* F. mosseae *AZ225C,* Rhizophagus irregularis *BEG141, foreign mixture-FMix (BEG12, AZ225C, and BEG141), local mixture-LMix of 14 AM fungal species belonging to five families, and mock-inoculated control] and Stage (one month, one year and two years of growth) as fixed factors and plots-within-replicates as a random factor. Three replicate plots per treatment.* | |
| *^b^In bold statistically significant values (*P *≤ 0.05).* | |

| **TABLE S3** \| *P*-values of three-way ANOVAs evaluating the effect of crop age (Age), arbuscular mycorrhizal fungal inoculant (Inoc) and cut (Cut) on plant growth and quality parameters of alfalfa (*Medicago sativa* L.). | | | | | | | | |
| --- | --- | --- | --- | --- | --- | --- | --- | --- |
| Treatment^a^ | Leaf dry weight | Stem dry weight | Shoot dry weight | Leaf/stem ratio | Forage N concentration | Forage P concentration | Forage N content | Forage P content |
| Age | **<0.001**^b^ | **<0.001** | **<0.001** | 0.731 | **<0.001** | **<0.001** | **<0.001** | **<0.001** |
| Inoc | **<0.001** | **<0.001** | **<0.001** | 0.512 | **<0.001** | **<0.001** | **<0.001** | **<0.001** |
| Cut | **<0.001** | **<0.001** | **<0.001** | **<0.001** | **0.001** | 0.406 | **0.042** | **<0.001** |
| Age x Inoc | **0.008** | **0.014** | **0.006** | 0.951 | **0.012** | 0.078 | **0.040** | **0.015** |
| Age x Cut | **0.040** | **<0.001** | **<0.001** | **<0.001** | **<0.001** | **<0.001** | **<0.001** | **<0.001** |
| Inoc x Cut | 0.369 | 0.134 | 0.227 | 0.391 | 0.818 | **0.002** | 0.533 | 0.103 |
| Age x Inoc x Cut | 0.086 | **0.036** | **0.038** | 0.771 | 0.787 | 0.306 | 0.051 | 0.055 |
| *^a^General mixed model including Age (1-year alfalfa in 2012 and 2-year alfalfa in 2013), Inoc [*Funneformis mosseae *BEG12,* F. mosseae *AZ225C,* Rhizophagus irregularis *BEG141, foreign mixture-FMix (BEG12, AZ225C, and BEG141), local mixture-LMix of 14 AM fungal species belonging to five families, and mock-inoculated control] and Cut (first and second cut) as fixed factors and plots-within-replicates as random effect. Three replicate plots per treatment.* | | | | | | | | |
| *^b^In bold statistically significant values (*P *≤ 0.05).* | | | | | | | | |

| **Table S4** \| *P*-values of three-way ANOVAs evaluating the effect of crop age (Age), arbuscular mycorrhizal fungal inoculant (Inoc) and cut (Cut) on palmitic acid (C16:0), stearic acid (C18:0), oleic acid (C18:1), linoleic acid (C18:2), linolenic acid (C18:3), and total fatty acid (TFA) concentration (g kg-1) and content of alfalfa (*Medicago sativa* L.) forage. | | | | | | | | | | | | | | | | | |  |
| --- | --- | --- | --- | --- | --- | --- | --- | --- | --- | --- | --- | --- | --- | --- | --- | --- | --- | --- |
|  |  |  |  |  |  |  |  |  |  |  |  |  |  |  |  |  |  |  |
| Treatment^a^ | C16:0 | |  | C18:0 | |  | C18:1 | |  | C18:2 | |  | C18:3 | |  | TFA | |  |
|  | g kg^-1^ | g m^-2^ |  | g kg^-1^ | g m^-2^ |  | g kg^-1^ | g m^-2^ |  | g kg^-1^ | g m^-2^ |  | g kg^-1^ | g m^-2^ |  | g kg^-1^ | g m^-2^ |  |
| Age | **<0.001**^b^ | **<0.001** |  | 0.193 | **<0.001** |  | **<0.001** | **<0.001** |  | **<0.001** | **<0.001** |  | **<0.001** | **<0.001** |  | **<0.001** | **<0.001** |  |
| Inoc | **<0.001** | **<0.001** |  | 0.513 | **<0.001** |  | **<0.001** | **<0.001** |  | **<0.001** | **<0.001** |  | **<0.001** | **<0.001** |  | **<0.001** | **<0.001** |  |
| Cut | 0.179 | **<0.001** |  | 0.354 | **0.002** |  | **0.008** | **<0.001** |  | **<0.001** | **<0.001** |  | **<0.001** | **<0.001** |  | **0.005** | **<0.001** |  |
| Age x Inoc | **0.004** | **0.025** |  | 0.345 | **0.046** |  | **<0.001** | **0.004** |  | **<0.001** | **0.013** |  | 0.159 | **0.007** |  | 0.085 | **0.008** |  |
| Age x Cut | 0.662 | **<0.001** |  | 0.282 | **0.002** |  | 0.431 | **<0.001** |  | **0.027** | **<0.001** |  | 0.250 | **<0.001** |  | 0.058 | **<0.001** |  |
| Inoc x Cut | **0.007** | 0.146 |  | 0.374 | 0.641 |  | **0.001** | 0.051 |  | 0.102 | 0.269 |  | **<0.001** | 0.247 |  | **<0.001** | 0.221 |  |
| Age x Inoc x Cut | 0.488 | **0.041** |  | 0.413 | 0.194 |  | 0.768 | **0.025** |  | 0.456 | **0.043** |  | 0.406 | **0.034** |  | 0.188 | **0.033** |  |
| *^a^General mixed model including Age (1-year alfalfa in 2012 and 2-year alfalfa in 2013), Inoc [*Funneformis mosseae *BEG12,* F. mosseae *AZ225C,* Rhizophagus irregularis *BEG141, foreign mixture-FMix (BEG12, AZ225C, and BEG141), local mixture-LMix of 14 AM fungal species belonging to five families, and mock-inoculated control] and Cut (first and second cut) as fixed factors and plots-within-replicates as random effect. Three replicate plots per treatment.* | | | | | | | | | | | | | | | | | |  |
| *^b^In bold statistically significant values (*P *≤ 0.05).* | | | | | | | | | | | | | | | | | |  |


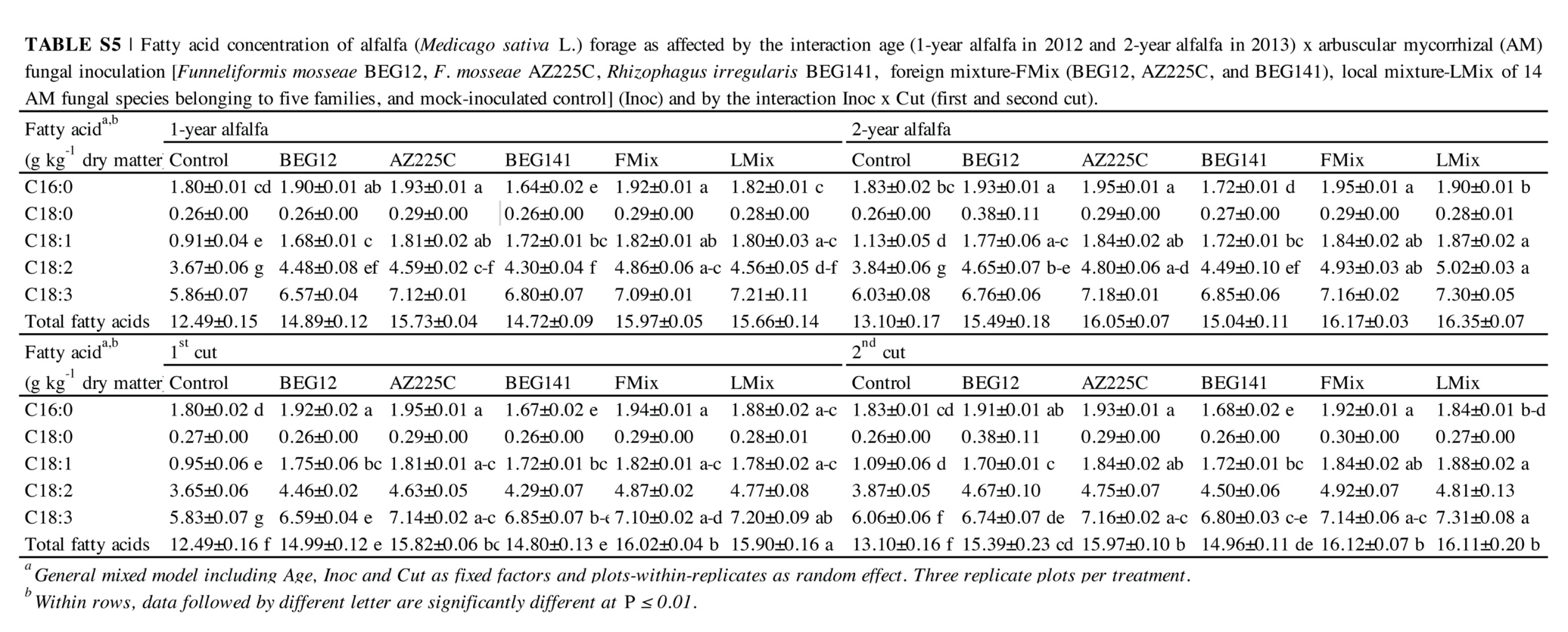


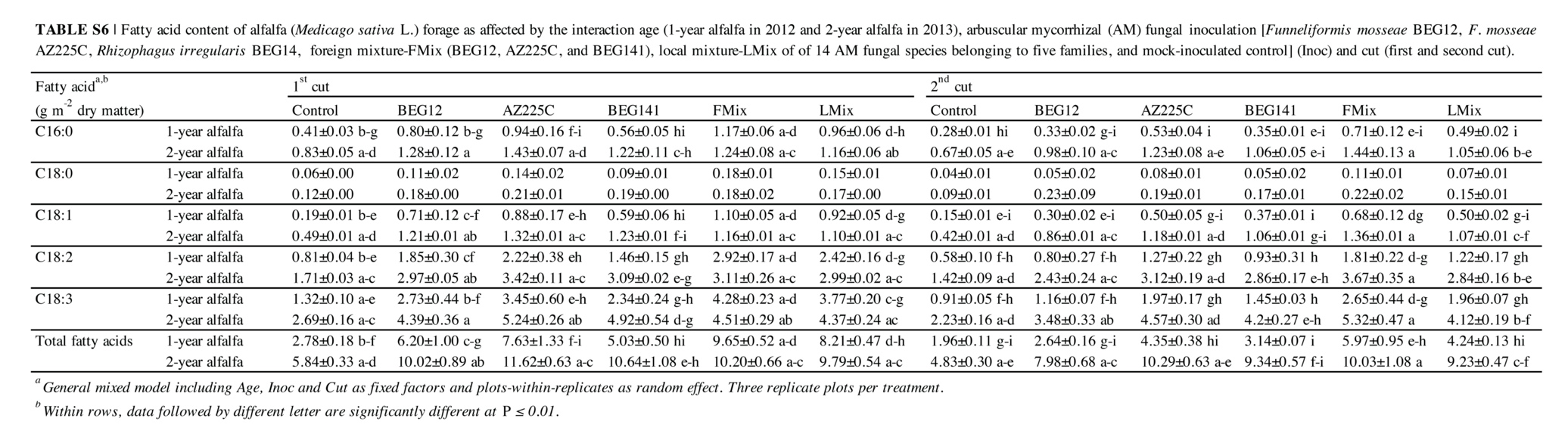


| **TABLE S7** \| Relative abundances of arbuscular mycorrhizal (AM) fungal phylotypes of *Funneliformis mosseae* and *Rhizophagus irregularis* retrieved at the beginning of spring growth in roots of 2-year alfalfa (*Medicago sativa* L.) inoculated by *F. mosseae* BEG12, *F. mosseae* AZ225C, *R. irregularis* BEG14, foreign mixture-FMix (BEG12, AZ225C, and BEG141), local mixture-LMix of seven AM fungal species, and control (mock-inoculated). Other species of Glomeromycotina are indicated as others. | | | | | | |
| --- | --- | --- | --- | --- | --- | --- |
| Phylotypes | Relative abundance in the roots (%) | | | |  |  |
|  | Control | BEG12 | AZ225C | BEG141 | FMix | LMix |
| BEG12 | - | 34.7 |  |  | 5.4 | - |
| AZ225C | - | - | 45.1 | - | 13.0 | - |
| BEG141 | - | - | - | 16.4 | 2.7 | - |
| Local *F. mosseae*^a^ | 44.0^**^ e | 25.6 cd | 20.9 bc | 7.4 a | 18.4 b | 29.5 d |
| Local *R. irregularis* cluster1 | 0.0 a | 2.2 ab | 2.6 ab | 1.9 ab | 4.3 b | 2.9 ab |
| Local *R. irregularis* cluster2 | 1.7 a | 2.3 a | 4.3 a | 1.3 a | 2.7 a | 3.9 a |
| Local *R. irregularis* cluster3 | 1.1 a | 2.8 a | 1.3 a | 1.7 a | 5.4 a | 3.9 a |
| Local *R. irregularis* cluster4 | 0.0 a | 3.4 b | 2.2 ab | 3.9 b | 2.7 ab | 2.9 ab |
| Local *R. irregularis* cluster5 | 6.9 a | 3.4 a | 3.9 a | 4.2 a | 2.2 a | 2.9 a |
| Local *R. irregularis* cluster6 | 6.4 b | 5.8 b | 3.1 ab | 1.9 a | 2.1 a | 1.9 a |
| Local *R. irregularis* cluster7 | 1.3 a | 2.3 a | 3.5 a | 1.7 a | 2.7 a | 2.4 a |
| Local *R. irregularis* cluster8 | 1.6 a | 3.4 a | 2.6 a | 3.4 a | 2.2 a | 1.9 a |
| Local *R. irregularis* cluster9 | 0.0 a | 3.4 a | 2.6 a | 1.3 a | 2.1 a | 2.9 a |
| Other | 37.0 b | 10.7 a | 7.9 a | 54.9 d | 34.1 b | 44.9 c |
| *^*^General linear model including Inoc as fixed factor. Three replicate plots per treatment.* | | | | | | |
| *^**^Within rows, data followed by different letter are significantly different at* P *≤ 0.01.* | | | | | | |

| **Table S8 \|** Results of marginal and sequential tests of the Distance-based linear model (DistLM) used for testing the significance of correlations between the arbuscular mycorrhizal fungal phylotypes retrieved in roots of 2-year alfalfa (*Medicago sativa* L.) inoculated by by *Funneliformis mosseae* BEG12, *F. mosseae* AZ225C, *Rhizophagus irregularis* BEG14, foreign mixture-FMix (BEG12, AZ225C, and BEG141), local mixture-LMix of seven AM fungal species, and control (mock-inoculated), and the plant trait pattern based on plant growth, N and P concentration, and fatty acid composition of forage at the beginning of spring growth. | | | | | |
| --- | --- | --- | --- | --- | --- |
| Marginal tests\Variable^a^ |  | SS(trace) | Pseudo-F | *P* | Proportion |
| Local *R. irregularis* cluster1 |  | 5058.2 | 15.5 | **0.004** | 0.492 |
| Local *R. irregularis* cluster9 |  | 3682.3 | 8.9 | **0.010** | 0.358 |
| Local *R. irregularis* cluster4 |  | 5023.5 | 15.3 | **0.003** | 0.489 |
| Local *R. irregularis* cluster8 |  | 1465.7 | 26.6 | 0.054 | 0.143 |
| Local *R. irregularis* cluster3 |  | 1518.9 | 2.8 | 0.053 | 0.149 |
| Local *R. irregularis* cluster2 |  | 272.0 | 0.435 | 0.727 | 0.026 |
| Local *R. irregularis* cluster7 |  | 1524.3 | 27.9 | **0.050** | 0.150 |
| Local *R. irregularis* cluster5 |  | 1560.7 | 28.6 | 0.057 | 0.152 |
| Local *R. irregularis* cluster6 |  | 2637.8 | 55.3 | **0.003** | 0.257 |
| Local *F. mosseae* |  | 2684.4 | 56.6 | **0.006** | 0.261 |
| Other Glomeromycotina sequences |  | 531.9 | 0.9 | 0.442 | 0.052 |
| *F. mosseae* inoculated BEG12 |  | 349.6 | 0.6 | 0.655 | 0.034 |
| *F. mosseae* inoculated AZ225C |  | 1346.7 | 24.1 | **0.049** | 0.131 |
| *R. irregularis*  BEG141 |  | 858.7 | 14.6 | 0.196 | 0.084 |
| Sequential tests\Variable | AICc | SS(trace) | Pseudo-F | *P* | R^2^/ Proportion |
| Local *R. irregularis* cluster1 | 106.9 | 5218.3 | 15.5 | **0.003** | 0.492 |
| *^a^DistLM based on step-wise selection and Akaike's information criterion (AICc). This analysis was done after having detected a significant relationship between the two matrices (AMF community vs. plant traits) (RELATE: ρ=0.46, P=0.4%; 999 permutations) and having found Local* R. irregularis *cluster 1 and cluster 6 as best phylotypes describing the relationship with plant traits by BEST analysis.* | | | | | |

## Supplementary Figures

**
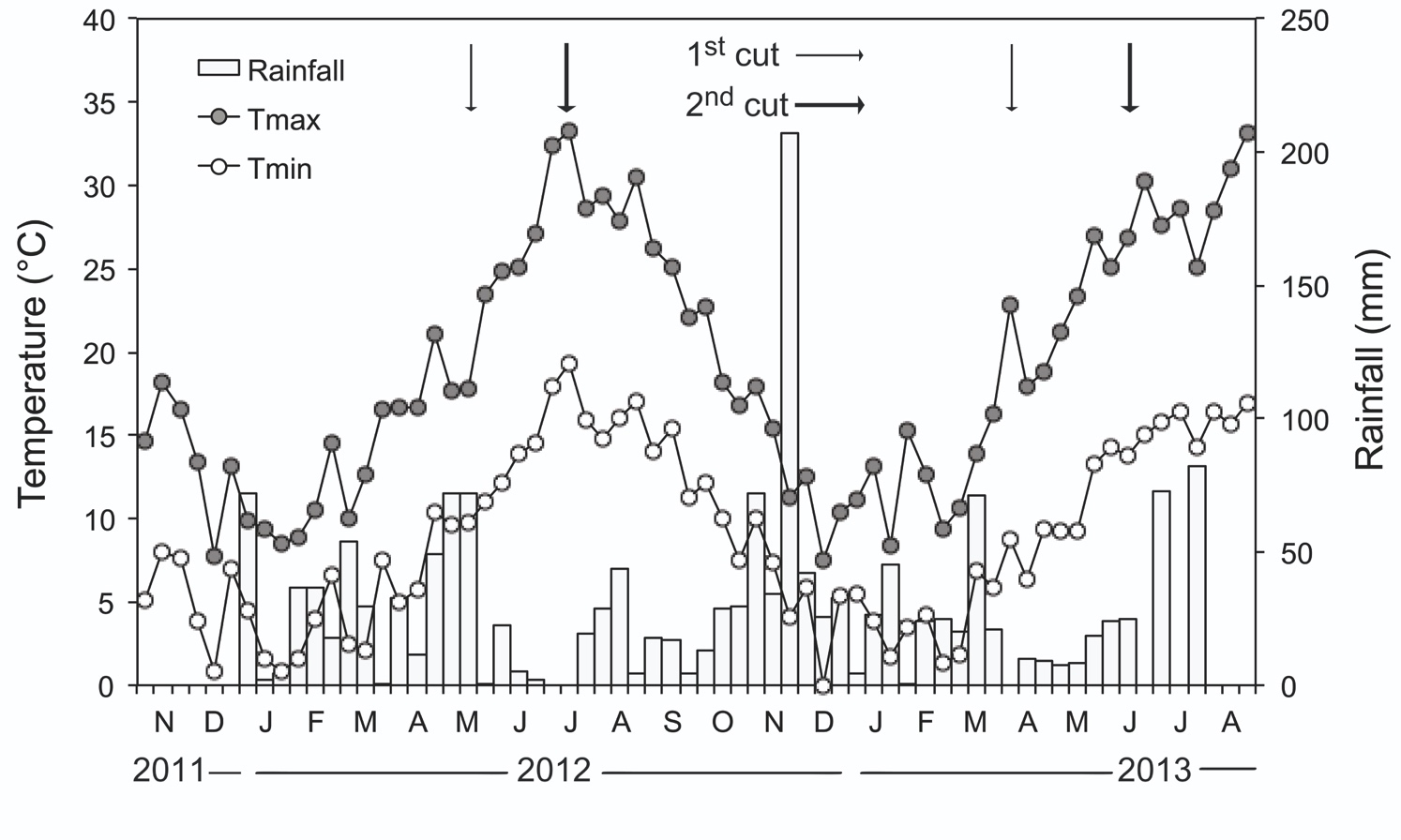
FIGURE S1 |** Decadal mean maximum and minimum temperatures (°C) and rainfall (mm) at the experimental site over the period October 2011 – August 2013. The arrows represent the time of 1^st^ and 2^nd^ cut in the one and two-year alfalfa (*Medicago sativa* L.) crop.


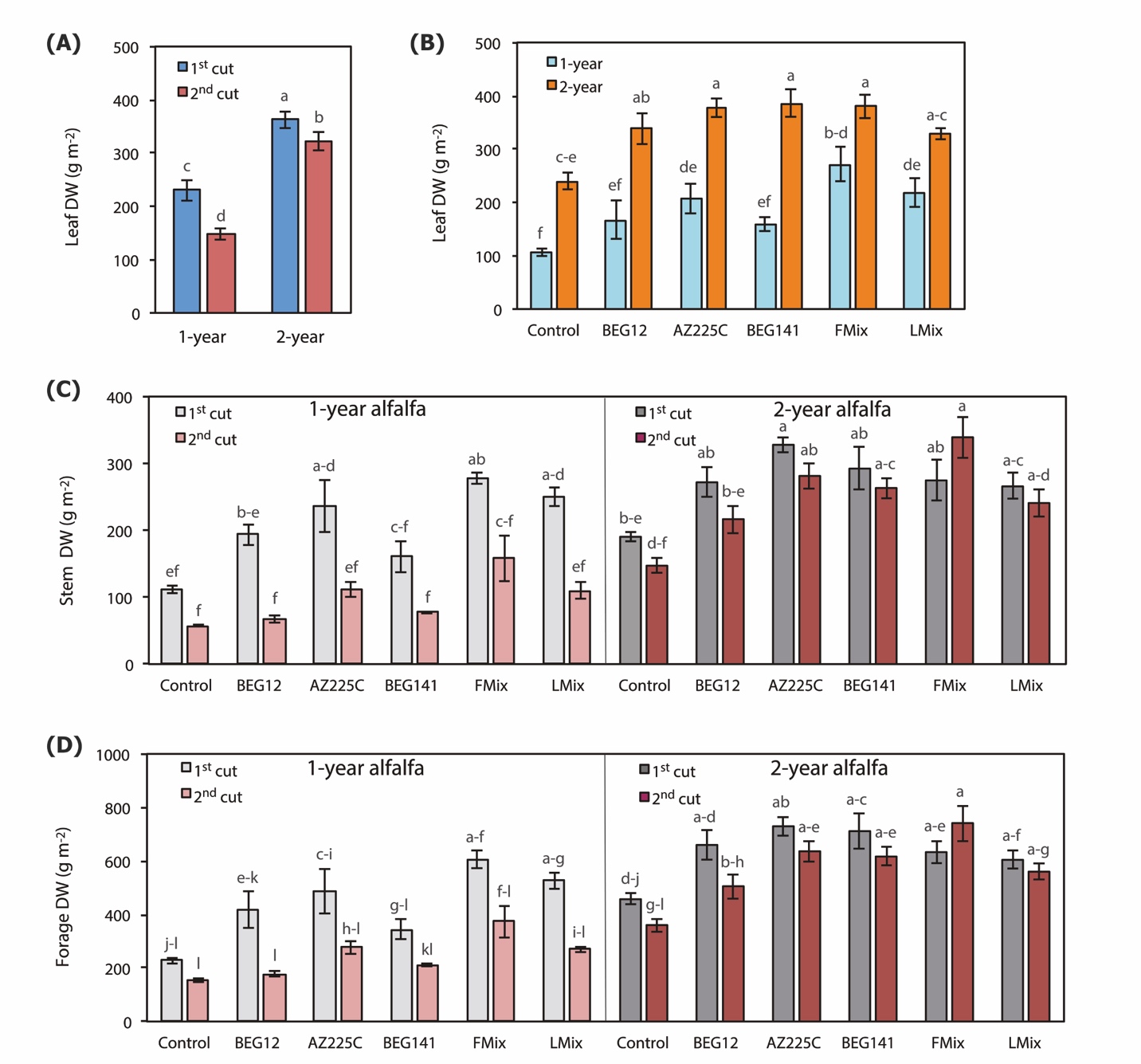


**FIGURE S2.** Leaf dry weight of alfalfa (*Medicago sativa* L.) as influenced by the interaction crop age (1-year and 2-year) and cut (1^st^ and 2^nd^ cut) (**A**) (n = 18), and by the interaction of Age and arbuscular mycorrhizal (AM) fungal inoculant [Inoc: *Funneliformis mosseae* BEG12, *Funneliformis mosseae* AZ225C, *Rhizophagus irregularis* BEG141, foreign mixture-FMix (BEG12, AZ225C, and BEG141), local mixture-LMix of 14 AM fungal species belonging to five families, and mock-inoculated control] (**B**) (n = 6), stem and forage dry weight as influenced by the interaction Age, Inoc and Cut (**C**, **D**) (n = 6). Data are mean ± standard error. Different letters indicate significant difference at *P* ≤ 0.036 (**Table S3**).

**
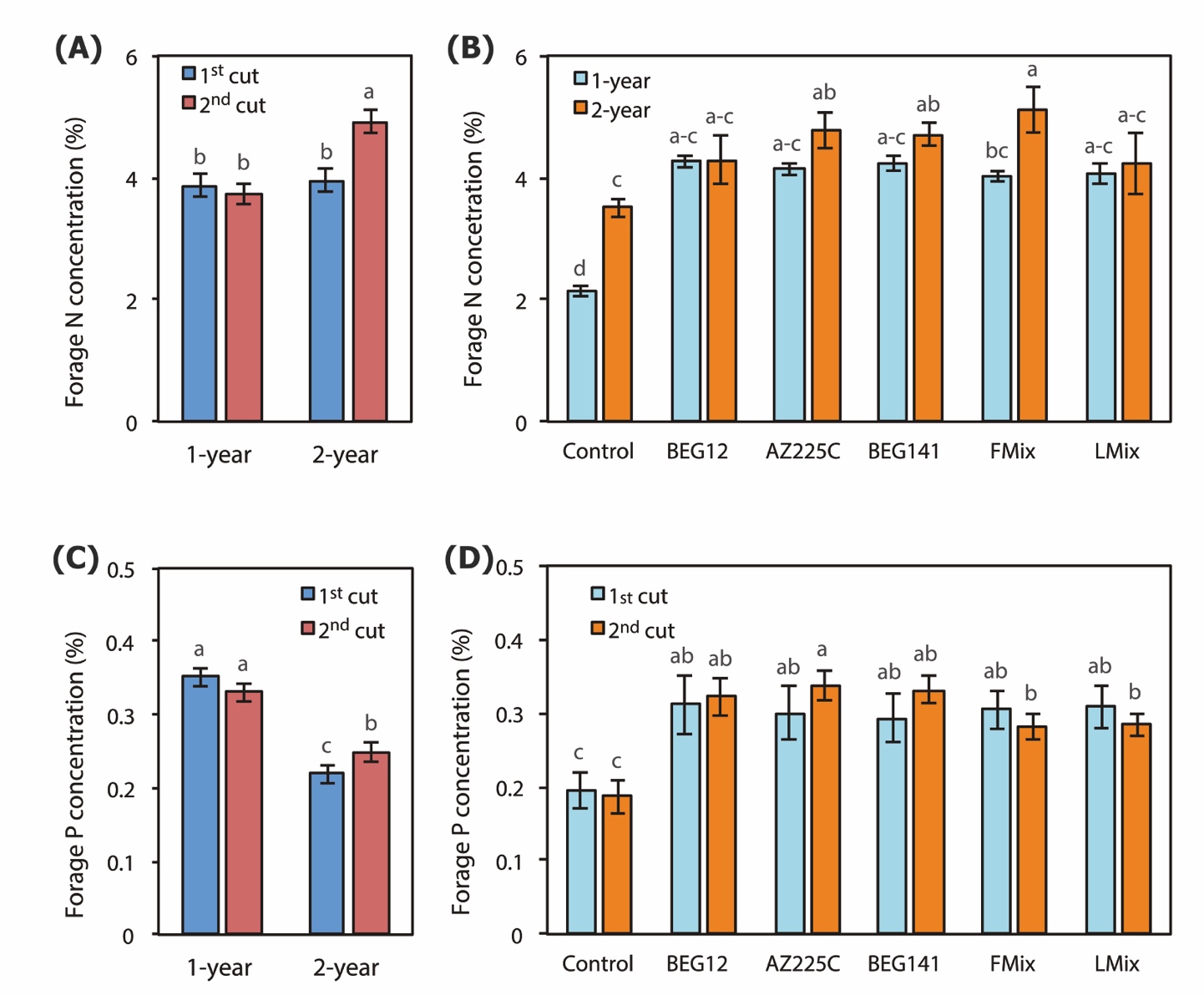
**

**FIGURE S3.** Forage N concentration of alfalfa (*Medicago sativa* L.) as influenced by the interaction crop age (1-year and 2-year) and cut (1^st^ and 2^nd^ cut) (**A**) (n = 18), and by the interaction Age and arbuscular mycorrhizal (AM) fungal inoculant [Inoc: *Funneliformis mosseae* BEG12, *Funneliformis mosseae* AZ225C, *Rhizophagus irregularis* BEG141, foreign mixture-FMix (BEG12, AZ225C, and BEG141), local mixture-LMix of 14 AM fungal species belonging to five families, and mock-inoculated control] (**B**) (n = 6), forage P concentration of alfalfa as influenced by the interaction Age and Cut (**C**) (n = 18), and by the interaction Inoc and Cut (**D**) (n = 6). Data are mean ± standard error. Different letters indicate significant difference at *P* ≤ 0.012 (**Table S3**).


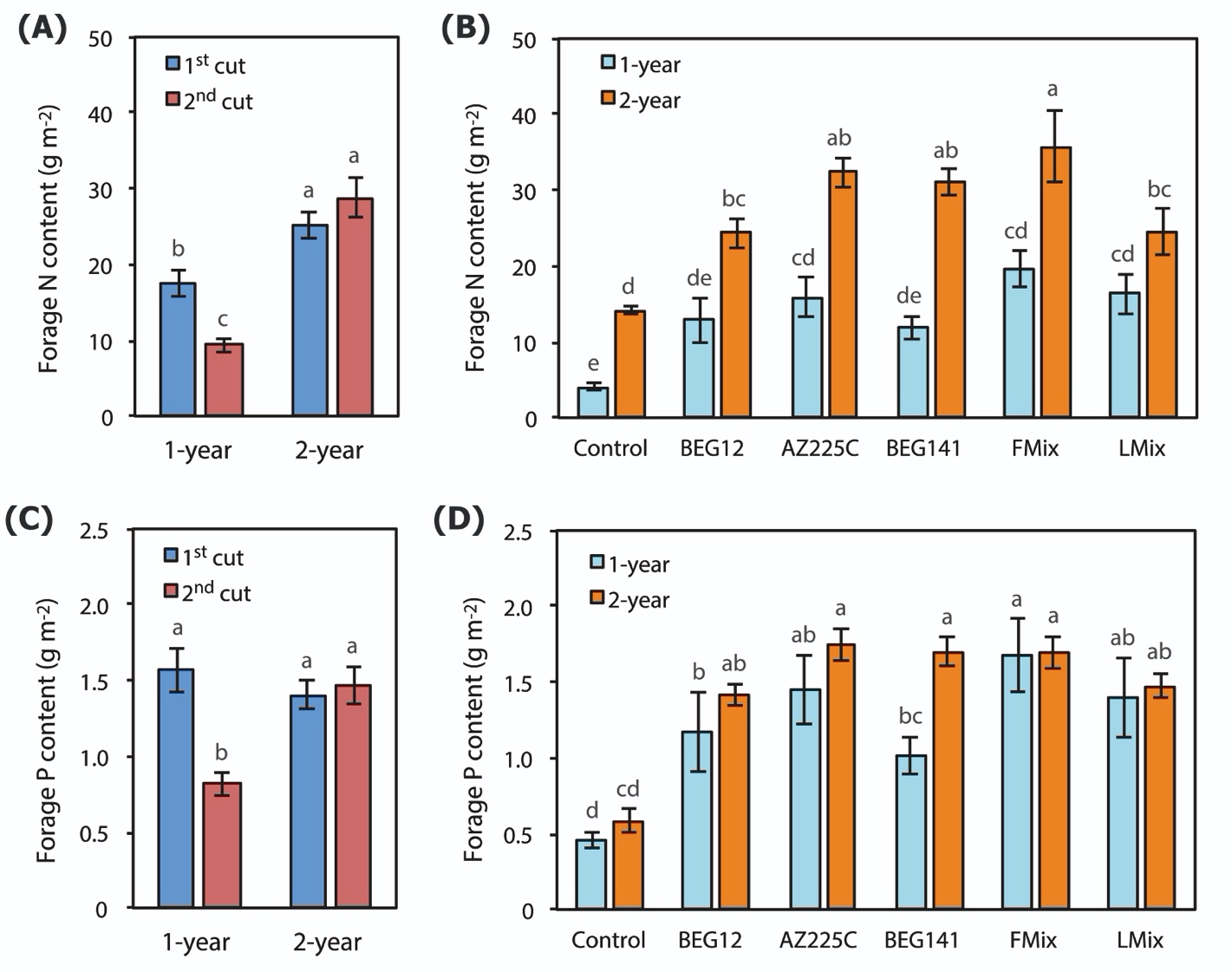


**FIGURE S4** **|** Forage N content of alfalfa (*Medicago sativa* L.) as affected by the interaction crop age (1-year and 2-year) and cut (1^st^ and 2^nd^ cut) (**A**), and by the interaction Age and arbuscular mycorrhizal fungal (AMF) inoculant [Inoc: *Funneliformis mosseae* BEG12, *Funneliformis mosseae* AZ225C, *Rhizophagus irregularis* BEG141, foreign mixture-FMix (BEG12, AZ225C, and BEG141), local mixture-LMix of 14 AM fungal species belonging to five families, and mock-inoculated control] (**B**); forage P content of alfalfa as affected by the interaction Age and Cut (**C**), and by the interaction Age and Inoc (**D**). Data are mean ± standard error. Different letters indicate significant difference at *P* ≤ 0.040 (**TABLE S2**).


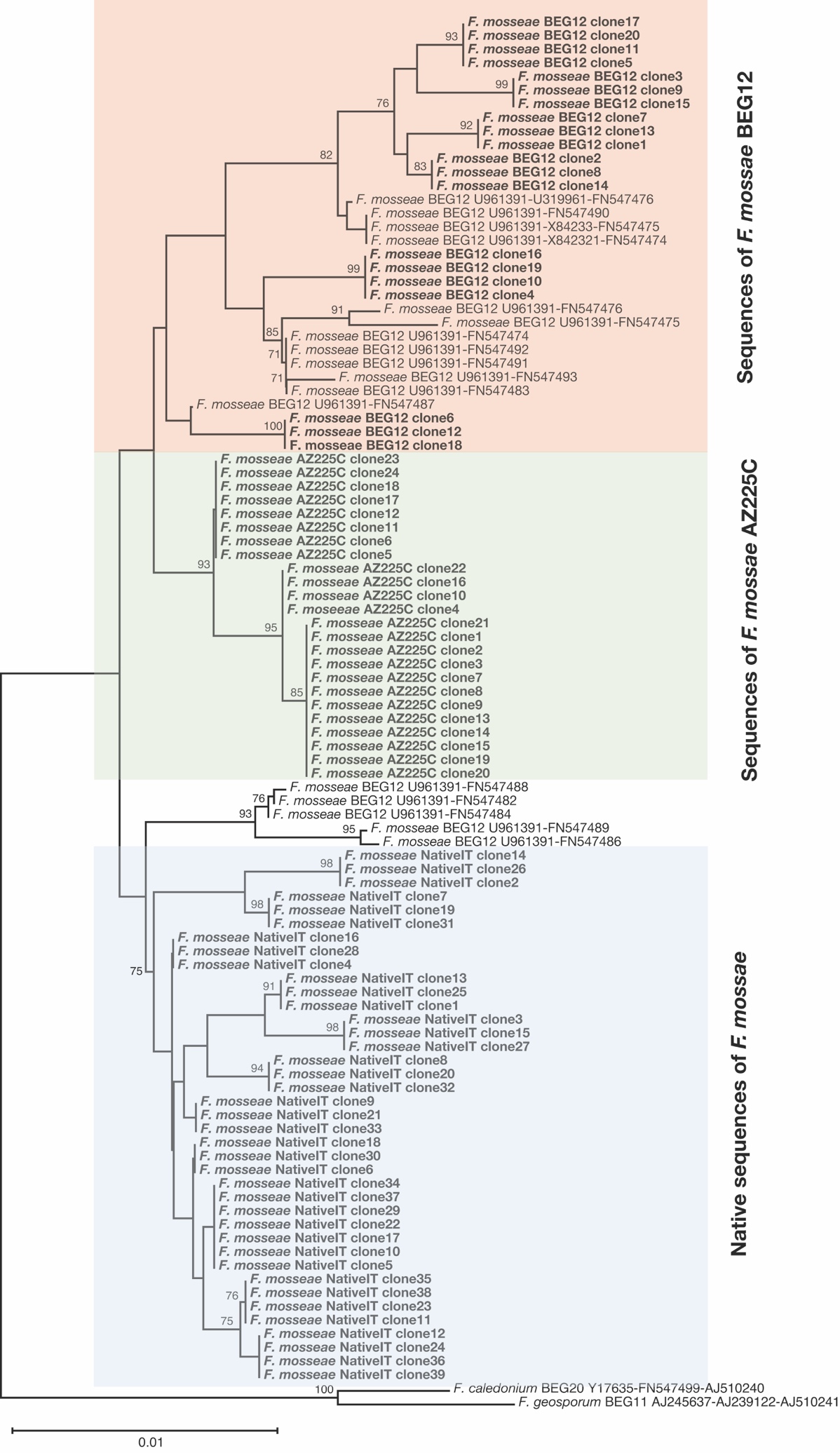


**FIGURE S5** **|** Neighbor-joining (NJ) tree of the nuclear ribosomal rDNA sequences of *Funneliformis mosseae,* originating from local strains and the non-native inoculated isolates BEG12 and AZ225C. The tree is composed by 20 and 24 newly generated sequences of BEG12 and AZ225C, respectively, 39 native *F. mosseae* sequences, plus 17 sequences of BEG12 as reference. The NJ tree is based on concatenated sequences of the small subunit ribosomal RNA gene (SSU; ca. 550 bp), the internal transcribed spacer 2 (ITS2; ca. 165 bp) and the large subunit ribosomal RNA gene (LSU; ca. 400 bp). Bootstrap values (based on 1000 replicates) are shown at the nodes. The scale bar indicates substitutions per site. Clades formed by sequences of native and inoculated *F. mosseae* strains are shown by colored rectangles. The concatenated sequences AJ245637-AJ239122-AJ5110241 of the isolate BEG11 of *Funneliformis geosporum* were used as outgroup. The newly generated sequences are highlighted in boldface.

**
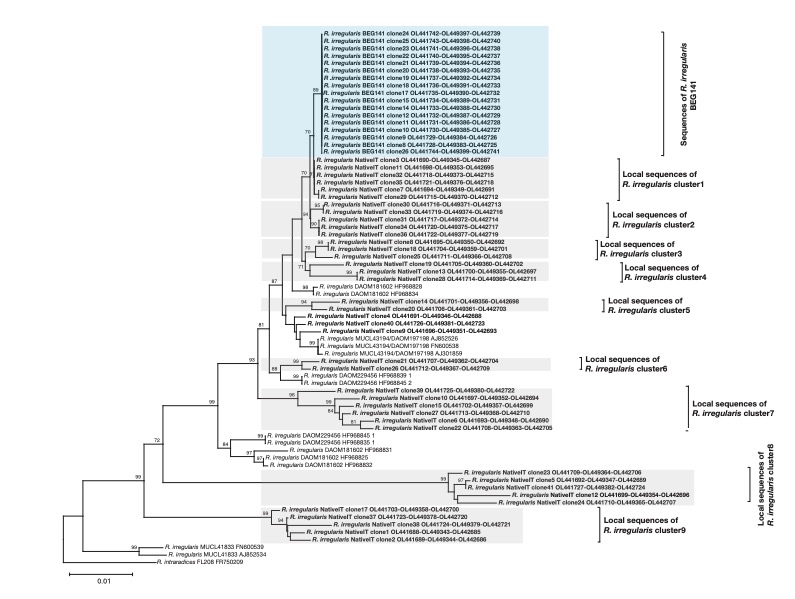
 FIGURE S6** **|** Neighbor-joining (NJ) tree of the nuclear ribosomal rDNA sequences of *Rhizophagus irregularis,* originating from local strains and the non-native inoculated isolates BEG141. The tree is composed by 17 newly generated sequences of BEG141 and 40 native *R. irregularis* sequences, plus 14 sequences of *R. irregularis* isolates as reference (DAOM181602, MUCL43194/DAOM197198, DAOM229456, MUCL41833). The NJ tree is based on concatenated sequences of the small subunit ribosomal RNA gene SSU (ca. 720 bp), 5.8S and the internal transcribed spacer 2 (ITS2) (ca. 430 bp), and the large subunit ribosomal RNA gene (LSU; ca. 365 bp). Bootstrap values (based on 1000 replicates) are shown at the nodes. The scale bar indicates substitutions per site. Clades formed by sequences of native and inoculated *R. irregularis* strains are shown by colored rectangles. The concatenated sequences FR750209-FM865565 of the isolate of *R. intraradices* FL208 were used as outgroup. The newly generated sequences are highlighted in boldface.

**
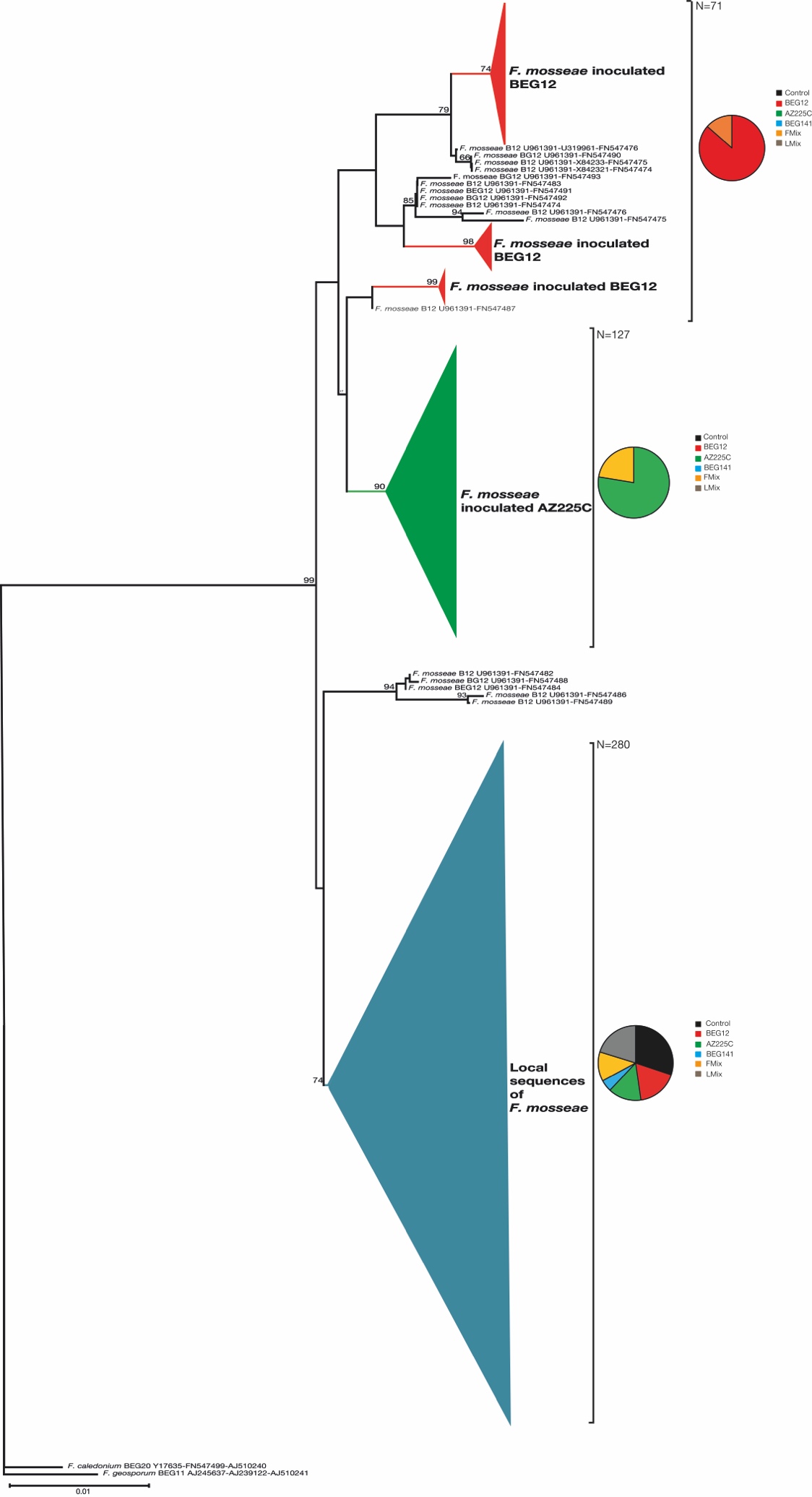
**

**FIGURE S7** **|** Neighbor-joining (NJ) collapsed tree of the nuclear ribosomal rDNA sequences of *Funneliformis mosseae* retrieved at the beginning of spring growth in roots of 2-year alfalfa (*Medicago sativa* L.) inoculated by *F. mosseae* BEG12, *F. mosseae* AZ225C, *R. irregularis* BEG14, foreign mixture-FMix (BEG12, AZ225C, and BEG141), local mixture-LMix of of 14 AM fungal species belonging to five families, and control (mock-inoculated). The tree is composed by 71 and 127 newly generated sequences of BEG12 and AZ225C, respectively, 280 native *F. mosseae* sequences, plus 17 sequences of BEG12 as reference. The NJ tree is based on concatenated sequences of the small subunit ribosomal RNA gene (SSU; ca. 550 bp), the internal transcribed spacer 2 (ITS2; ca.165 bp) and the large subunit ribosomal RNA gene (LSU; ca. 400 bp). Bootstrap values (based on 1000 replicates) are shown at the nodes. The scale bar indicates substitutions per site. Clades formed by sequences of native and inoculated *F. mosseae* strains are shown by colored branches and triangles. Pie charts represent the relative abundances of phylotypes in the inoculation treatments. The concatenated sequences AJ245637-AJ239122-AJ5110241 of the isolate BEG11 of *Funneliformis geosporum* were used as outgroup. The newly generated sequences are highlighted in boldface.

**
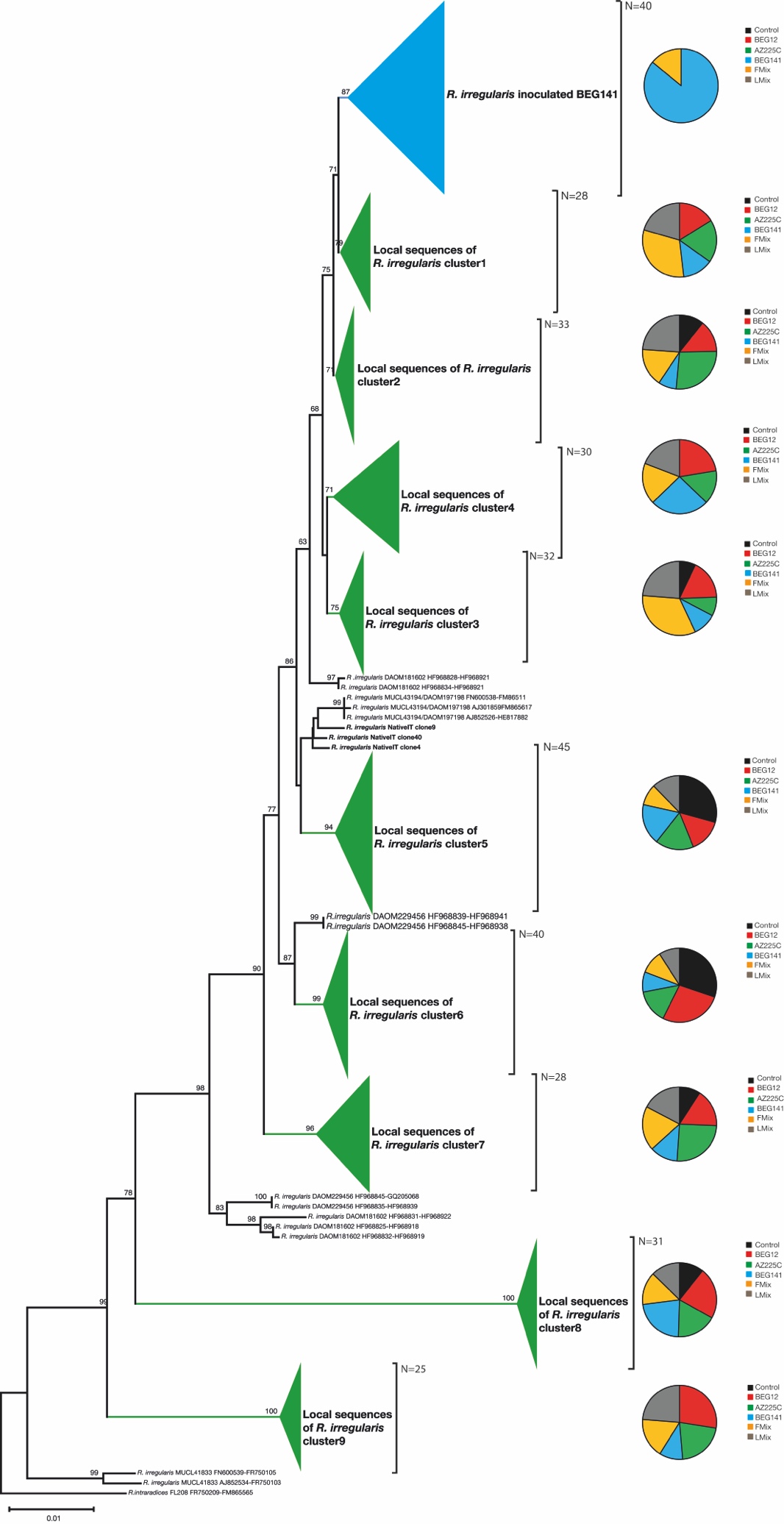
**

**FIGURE S8** **|** Neighbor-joining (NJ) collapsed tree of the nuclear ribosomal rDNA sequences of *Rhizophagus irregularis* retrieved at the beginning of spring growth in roots of 2-year alfalfa (*Medicago sativa* L.) inoculated by *Funneliformis mosseae* BEG12, *F. mosseae* AZ225C, *R. irregularis* BEG14, foreign mixture-FMix (BEG12, AZ225C, and BEG141), local mixture-LMix of 14 AM fungal species belonging to five families, and control (mock-inoculated). The tree is composed by 40 newly generated sequences of BEG141 and 291 native *R. irregularis* sequences, plus 14 sequences of *R. irregularis* isolates as reference (DAOM181602, MUCL43194/DAOM197198, DAOM229456, MUCL41833). The NJ tree is based on concatenated sequences of the small subunit ribosomal RNA gene on concatenated sequences of the small subunit ribosomal RNA gene SSU (ca. 720 bp), 5.8S and the internal transcribed spacer 2 (ITS2; ca. 430 bp), and the large subunit ribosomal RNA gene (LSU; ca. 365 bp). Bootstrap values (based on 1000 replicates) are shown at the nodes. The scale bar indicates substitutions per site. Clades formed by sequences of native and inoculated *R. irregularis* strains are shown by colored branches and triangles. Pie charts represent the relative abundances of phylotypes in the inoculation treatments. The concatenated sequences FR750209-FM865565 of the isolate of *R. intraradices* FL208 were used as outgroup. The newly generated sequences are highlighted in boldface.

**2. Supplementary Results**

**2.1 Plant growth, forage yield and N and P content**

Leaf dry weight (leaf DW) was influenced by the interactions age x cut and age x inoculant (Table S3). Averaged over AMF inoculants, leaf DW was higher in the 2-year crop compared with the 1-year one and consistently higher in the 1^st^ cut compared with the 2^nd^ one (Figure S2A). The increase across years of the 2^nd^ cut was higher than the one of the 1^st^ cut. Averaged over cuts, leaf DW under all treatments increased over time, and in the 1-year crop was significantly higher than control with AZ225C, Lmix and Fmix, while did not change with BEG12 and BEG141 (Figure S2B). Conversely, in the 2-year crop leaf DW with all inoculants was significantly higher than control.

Stem dry weight (stem DW) was influenced by the interaction age x inoculation x cut (Table S3 and Figure S2C). Differences among control and inoculants and within inoculants become noticeable in the 2-year crop: the highest stem DW was recorded with AZ225C in the 1^st^ cut, and with Fmix in the 2^nd^ cut.

Forage dry weight (forage DW) showed a similar pattern to stem DW across years and cuts (Figure S2D). It increased from the 1-year to the 2-year crop and decreased from the 1^st^ cut to the 2^nd^ cut, but the difference between cuts was higher in the 1-year crop. Inoculants differentially influenced forage DW: in the 1-year crop, values of the 1^st^ cut of alfalfa inoculated with Fmix, Lmix and AZ225C were significantly higher than control, while at the 2^nd^ cut they did not change with inoculants. In the 2-year crop, values of the 1^st^ cut of alfalfa inoculated with AZ225C and BEG141 were significantly higher than control, while at the 2^nd^ cut the increases were significant only with AZ225C, BEG141 and Fmix.

Forage N concentration was influenced by the interactions age x cut and age x inoculant (Table S3). Averaged over AMF inoculants, forage N concentration did not vary between cuts of the 1-year crop and in the 1^st^ cut of the 2-year crop, and it was by 27% higher in the 2^nd^ cut of the 2-year crop (Figure S3A). Averaged over cuts, forage N concentration of the 1-year crop was higher with all inoculants than in the control, whereas differences among inoculants were not significant (Figure S3B). By contrast, in the 2-year crop, forage N concentration in plants increased compared with the 1-year crop only in control and in Fmix plots. The highest forage N concentration was recorded with Fmix at 2 year of growth. Forage P concentration was influenced by the interactions age x cut and cut x inoculant (Table S3). Averaged over AMF inoculants, forage P concentration was higher in the 1-year crop than in the 2-year crop, and values of cuts were similar in the 1-year crop and slightly higher in the 2^nd^ cut than in the 1^st^ cut in the 2-year crop (Figure S3C). Averaged over crop ages, forage P concentration was increased by AMF inoculation in both cuts, and the effect was similar with all inoculants (Figure S3D).

Forage N and P contents were influenced by the interactions age x cut and age x inoculant (Table S3). Averaged over AMF inoculants, forage N content increased from the 1-year to the 2-year crop in both cuts, but value of the 1^st^ cut was higher than the one of the 2^nd^ cut in the 1-year crop, and did not change between cuts in the 2-year crop (Figure S4A). Averaged over cuts, forage N content increased with crop age (Figure S4B). Compared with uninoculated control, forage N content was significantly increased only by AZ225C, Fmix and Lmix in the 1-year crop, while it was increased by all inoculation treatments in the 2-year crop. Averaged over AMF inoculants, forage P content was similar in both years and cuts, except for the 2^nd^ cut of the 1-year crop that was 45% lower (Figure S4C). Averaged over cuts, forage P content was increased by inoculation treatments in both years (Figure S4D). The highest values were recorded with Fmix in both years and with AZ225C and BEG141 only in the second year.
